# A Machine Learning-Aided Equilibrium Model of VTSA Processes for Sorbents Screening Applied to CO_2_ Capture from Diluted Sources

**DOI:** 10.1021/acs.iecr.2c01695

**Published:** 2022-09-06

**Authors:** Alexa Grimm, Matteo Gazzani

**Affiliations:** † Utrecht University, Copernicus Institute of Sustainable Development, Princetonlaan 8a, 3584 CBUtrecht, The Netherlands; ‡ Sustainable Process Engineering, Chemical Engineering and Chemistry, Eindhoven University of Technology, 5612 APEindhoven, The Netherlands

## Abstract

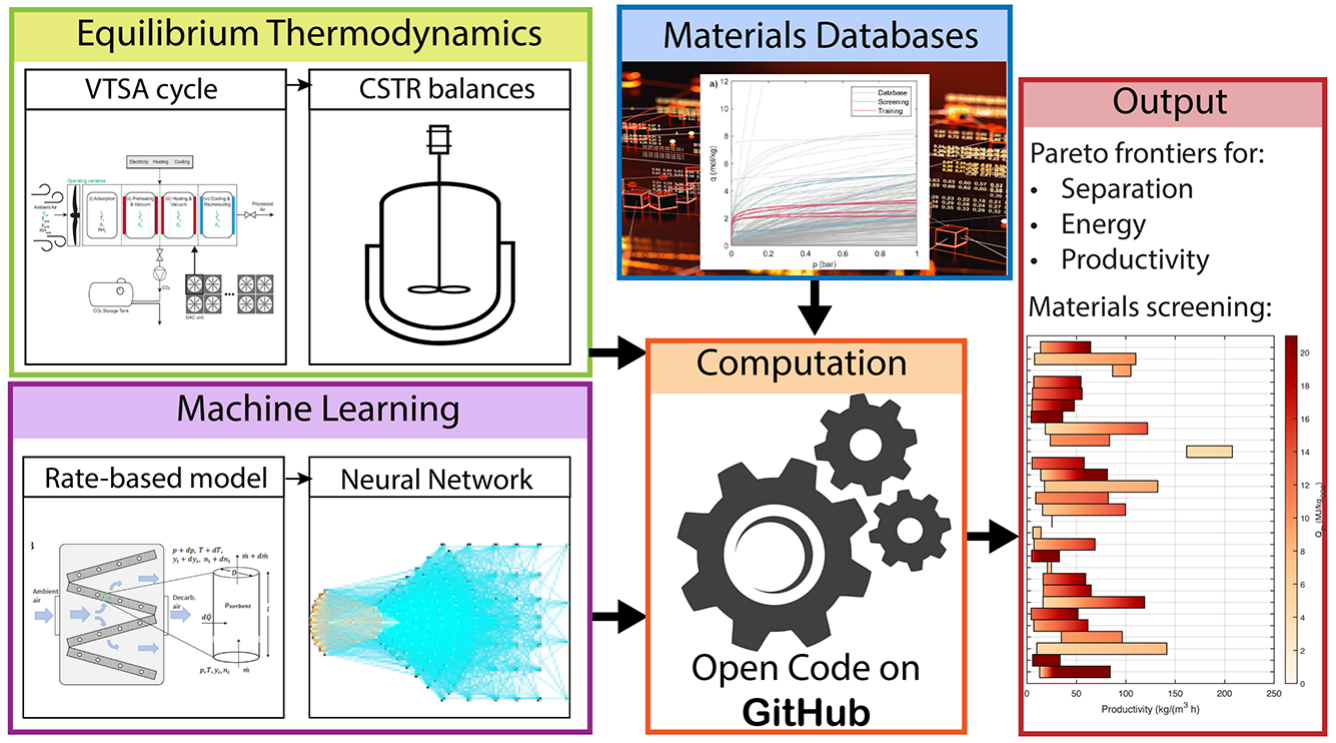

The large design space of the sorbents’ structure and the associated capability of tailoring properties to match process requirements make adsorption-based technologies suitable candidates for improved CO_2_ capture processes. This is particularly of interest in novel, diluted, and ultradiluted separations as direct CO_2_ removal from the atmosphere. Here, we present an equilibrium model of vacuum temperature swing adsorption cycles that is suitable for large throughput sorbent screening, e.g., for direct air capture applications. The accuracy and prediction capabilities of the equilibrium model are improved by incorporating feed-forward neural networks, which are trained with data from rate-based models. This allows one, for example, to include the process productivity, a key performance indicator typically obtained in rate-based models. We show that the equilibrium model reproduces well the results of a sophisticated rate-based model in terms of both temperature and composition profiles for a fixed cycle as well as in terms of process optimization and sorbent comparison. Moreover, we apply the proposed equilibrium model to screen and identify promising sorbents from the large NIST/ARPA-E database; we do this for three different (ultra)diluted separation processes: direct air capture, *y*_CO_2__ = 0.1%, and *y*_CO_2__ = 1.0%. In all cases, the tool allows for a quick identification of the most promising sorbents and the computation of the associated performance indicators. Also, in this case, outcomes are very well in line with the 1D model results. The equilibrium model is available in the GitHub repository https://github.com/UU-ER/SorbentsScreening0D.

## Introduction

In Samuel Beckett’s play “Waiting for Godot”,^53^ two characters, Vladimir and Estragon, manage to keep the audience’s attention while nothing happens: indeed, Godot never arrives. Few would disagree that the play somehow represents the story of CO_2_ capture and storage (CCS): since the early 2000s, CCS has taken on a role in key climate mitigation technology but has so far failed to deliver the required CO_2_ capture capacity. However, as we keep burning fossil fuels to match the increasing energy demand,^1^ a timely and cost-effective decarbonization will be relying more and more on CCS.^2^ In order to limit global warming to 2 °C, the amount of CO_2_ captured needs to increase from the current 40 Mt per year to 1070 in 2030 and 7600 in 2050.^1^ Notably, 70 Mt per year in 2030 and 630 Mt per year in 2050 need to be removed from the air using engineered technologies, for example, via direct air capture (DAC). While these figures may seem daunting, it is clear that the role of CCS in the energy system is becoming more and more essential. Even if the under performance of CCS is just remotely connected to technical reasons, one could say that CO_2_ separation processes are complex but also offered commercially with warranties by a few companies, it is important to keep improving CO_2_ capture technologies; the scale of deployment simply asks us for it. This is particularly true when considering gases with (ultra)diluted CO_2_ concentrations, e.g., DAC. Not surprisingly, the academic and industrial interest in DAC is growing significantly.^3−9^ If we look at the DAC industrial and scientific landscape, separation based on solid sorbents has so far attracted most of the attention and has been successfully demonstrated at the relevant scale.^10,11^ Moreover, significant scientific efforts are directed toward the development of new, better performing sorbents for DAC.^9,12,13^ At the same time, adsorption-based technologies are being developed and researched for CO_2_ capture from point sources, from flue gas to syngas to steel work gases.^14,15^

While several studies investigate, from a process and material perspective, the development of better performing sorbents for CO_2_ capture from point sources, there are no works, to the best of our knowledge, that do so in the realm of diluted CO_2_ sources, e.g., DAC.

The choice of the adsorbent is indeed a key factor for the optimal design of a capture process, and several research groups have developed computational techniques to design new sorbents and to characterize their thermodynamic (and transport) properties.^16−18^ Hundreds of thousands of theoretical sorbent materials have been simulated and could in principle be synthesized, provided the right experimental processes are available and the theoretical crystal is stable. On the other hand, the availability of all these theoretical materials requires a suitable screening procedure, which needs to be fast and accurate enough to provide a reliable ranking. To this end, different approaches exist. A first, simple approach is the calculation of characteristic parameters, like the working capacity or the heat of adsorption, on the basis of the isotherm data of the materials.^19−21^ While this analysis can be extremely fast, the results only provide a rough overview about the suitability of the materials. For a more reliable understanding, a process-based analysis is required.^22−25^ Ideally, for every sorbent, a detailed process simulation combined with process optimization is carried out; however, such a framework is computationally expensive and may take up to several days per sorbent.^26,27^ Two main alternatives exist to speed up the screening. On the one hand, a rigorous process simulation can be coupled to machine learning techniques, for example, in the convergence to cyclic steady state. Recently, Pai et al.^28^ developed a generalized data-driven surrogate model that well reproduces a PSA/VSA process. The framework makes use of a dense feed forward neural network and can significantly reduce the simulation and optimization time while showing a high accuracy. The data-driven model is trained using the simulation results of different sorbents and operating conditions and can be used as a screening tool, as long as the CO_2_ and N_2_ adsorption isotherm of the material can be described by the implemented numerical adsorption model. This approach shows great potential for bridging the simulation, optimization, and sorbent screening; however, it requires a large representable data set for training and testing the algorithms. The second, more traditional approach is to simplify rate-based process simulations using equilibrium. In this case, simpler models are used to solve the material and energy balances. A few key works are available in the literature that demonstrate the potential of equilibrium-based simulations. In their seminal work, Maring and Webley^29^ developed a simplified pressure/vacuum swing adsorption (P/VSA) model for a binary mixture. They adopted a well-mixed bed approach for the cycle, consisting of three steps: blow-down, repressuration, and adsorption. To further simplify the model, they assumed adiabatic operation and equilibrium between the adsorbed and gas phases. In addition, they proposed an approach to directly calculate the cyclic steady state (CSS). The model was validated for postcombustion CO_2_ capture by VSA against rate-based numerical simulations; four different types of sorbents were tested. More recently, Subramanian Balashankar et al.^30^ expanded the approach of Maring and Webley^29^ by treating the process as isothermal and by considering different VSA cycle configurations, which included blow-down, evacuation, pressurization, and adsorption steps. Notably, the model was used to screen 197 adsorbents from the NIST/ARPA-E database for CO_2_ capture application. When looking at the temperature swing adsorption (TSA) landscape, Joss et al.^31^ developed a shortcut model for a four step TSA cycle and binary mixture. No spatial gradients were considered in the model, and the partial differential equations were reduced to ordinary differential equations. In addition, the model directly calculates the CSS semi-analytically, which reduces the computational complexity. More recently, Ajenifuja et al.^32^ further developed the work of Maring and Webley^29^ and Joss et al.^31^ and presented an equilibrium model to quickly scan adsorbents using a three-step TSA cycle. Instead of using partial differential equations, a set of nonlinear algebraic equations is used for mass and energy balances, which reduces the computational time. The methodology is applied to screen 75 adsorbents for the capture of CO_2_.

With this work, we further contribute to the topic of equilibrium-based modeling tools for computationally efficient analysis of adsorption processes. The framework we present builds upon the excellent works discussed above and further extends them by

- Bridging the equilibrium-based approach to machine learning; i.e., we improve the accuracy and prediction capability of an equilibrium model using neural networks. This allows, for example, us to include the productivity as key performance indicator and to consider saturation levels in the bed during adsorption below 100%.
- Modeling a vacuum-temperature swing adsorption cycle; i.e., we add the vacuum step to the TSA cycle.
- Considering a ternary mixture as feed where CO_2_ is not necessarily the most retained gas; i.e., we add H_2_O adsorption, which is often the most adsorbed species in CO_2_ capture with V/TSA.
- Including multiple CO_2_ isotherm types in the model, i.e., Toth, extended Toth model (Toth-cp), Langmuir–Freundlich, dual-site Langmuir (DSL), and s-shaped isotherm model.
- Applying the tool to dilute or ultradilute CO_2_ concentrations, i.e., from CO_2_ capture from air to flue gas with 1% CO_2_.

The model that we present here is benchmarked with a well-established detailed 1D VTSA model. Furthermore, we apply the proposed method to efficiently scan the NIST/ARPA-E Database of Novel and Emerging Adsorbent Materials (NIST-ISODB) in search of promising sorbents to capture CO_2_. The NIST-ISODB database is the world’s largest public collection of experimental gas adsorption isotherms.^33^ It includes over 30 000 isotherms for a wide range of adsorbent materials including MOFs, COFs, zeolites, activated carbons, and amorphous porous polymers and serves as basis for several data-driven analyses.^34,35^ In addition, we complement the NIST database with adsorbents data from publications that have not been included yet.^22,36,37^

This paper is organized as follows: in Equilibrium Model for VTSA, we describe the 4 step VTSA process and the mathematical modeling framework of the 0D model. In addition, an overview of the key performance indicators is given. In Model Validation, the 0D model is validated against the rate-based model by comparing the performance for a specific simulation (e.g., in terms of time steps and temperatures) as well as optimizing the results. Finally, in the Sorbents Screening section, the model is applied for the screening of more than 2100 materials for CO_2_ capture from diluted sources.

## Equilibrium Model for VTSA

**Figure 1:**
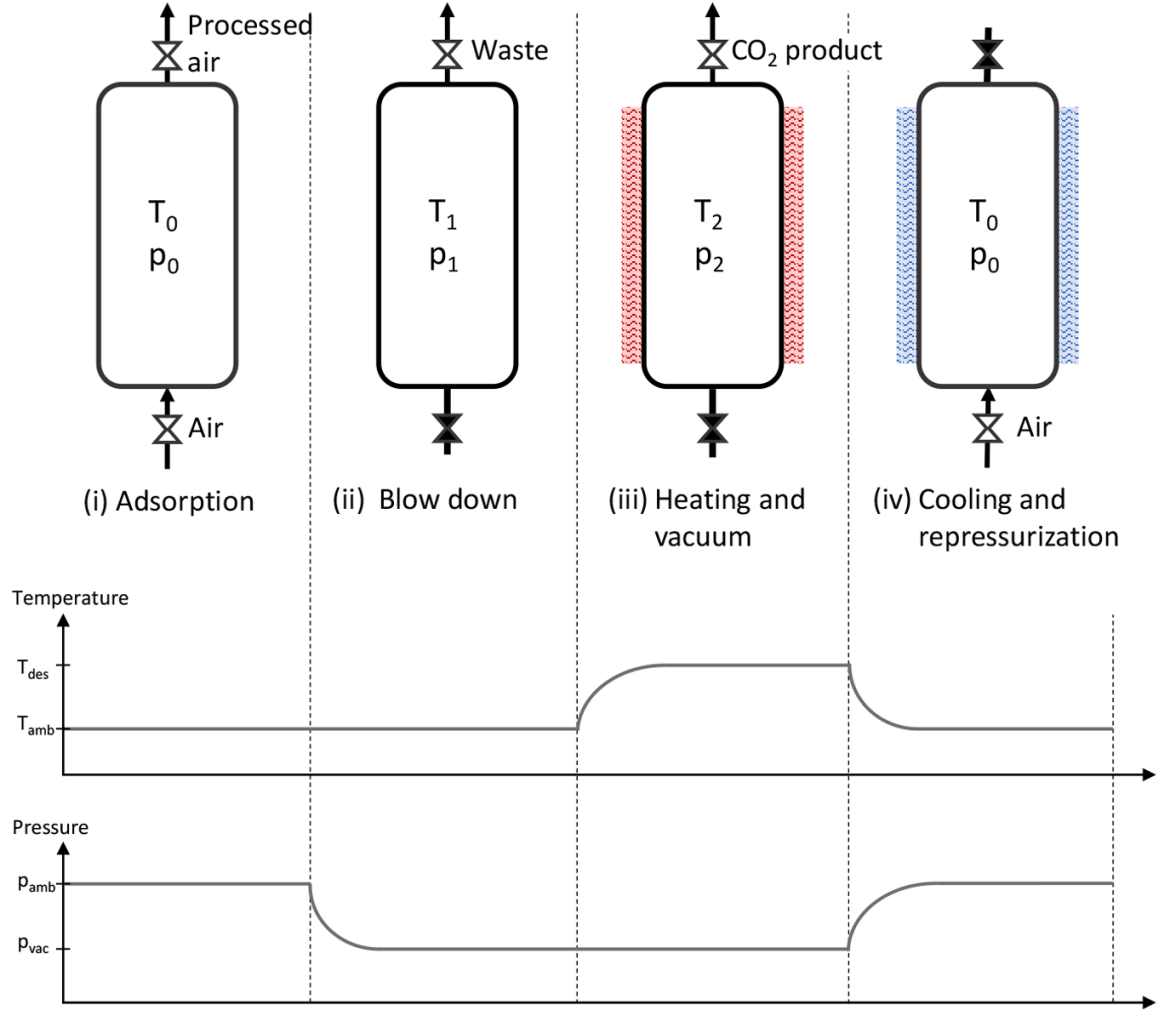
Simplified VTSA cycle. Indirect heating and cooling are performed with an open end or open entrance, respectively. The temperature and pressure profiles are indicative.

The VTSA cycle considered in this work is shown in Figure 1 and consists of four steps: adsorption, blow-down, heating, and cooling. This is a slightly simplified version (i.e., no preheating step) of the VTSA cycle adopted for CO_2_ capture from air in Sabatino et al.^37^ Moreover, we consider a feed stream consisting of three components, i.e., CO_2_, H_2_O, and N_2_, where CO_2_ and H_2_O can adsorb, while N_2_ is treated as an inert. It is worth noting that the model can be adapted to consider additional gas species. Different from other simplified models,^29,30,32^ the targeted species is not necessarily the strongly adsorbed one but can also be the weakly adsorbed component; i.e., H_2_O typically shows a higher adsorption capacity than CO_2_ when using materials of interest for CO_2_ capture from diluted streams.

The model builds upon the approach presented in previous works,^29,30,32^ where the key model assumptions are (i) the bed is treated as a well-stirred reactor, (ii) the gas and the solid phases are in equilibrium during all steps of the cycle, (iii) the gas phase behaves like a perfect gas, and (iv) the pressure drop in the bed as well as (v) heat transfer resistances are negligible. Accordingly, at any time instant, the total amount of moles of component *i* in the bed *N*_*i*,total_ is calculated from the number of moles in the solid (s) and in the fluid (f) phase
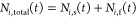
1with
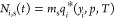
2
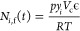
3where *p* is the pressure in the column, *y*_*i*_ is the mole fraction of species *i*, *V*_c_ is the column volume, ϵ is the void fraction, *R* is the universal gas constant, *T* is the temperature, *m*_s_ is the mass of the adsorbent, and , the equilibrium adsorbed amount.  can be calculated from any suitable isotherm; in this work, we have implemented multiple isotherm equations so as to include in the screening as many materials from the NIST database as possible: Toth, extended Toth (Toth-cp), Langmuir–Freundlich, dual site-Langmuir (DSL), and s-shaped isotherms. The detailed equations can be found in Table S12. The overall material balance considering the column and the flows entering/leaving can be written as
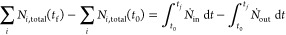
4

The material balance is complemented by the energy balance, which can be written as
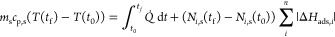
5where  is considered positive when entering. The isosteric heat Δ*H*_ads,*i*_ is calculated using the Clausius–Clapeyron equation:
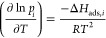
6

It is worth noting that the specific heat capacities of the gases are considered negligible with respect to the specific heat of the solid material and, therefore, excluded from the energy balance.

**Figure 2:**
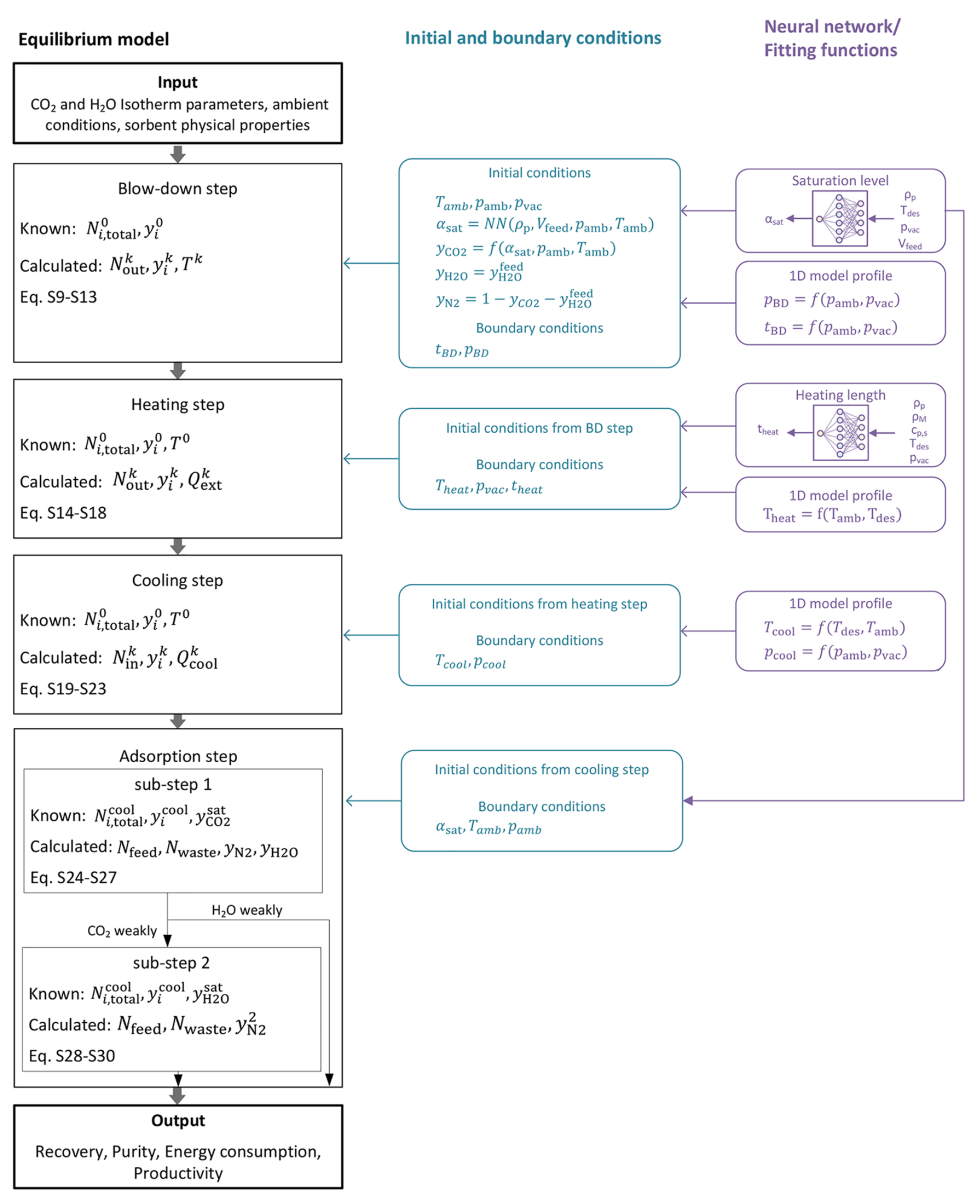
Architecture of the equilibrium model.

In the following, we discuss how the material and energy balances are written and solved for each step in the cycle. The overall resolution strategy with known/unknown variables is also shown in Figure 2. A more detailed list of the equations used in each step (derived from the previous balances) are reported in Table S5. Similar to previous works, the starting point of the 0D simulation is the end of the adsorption, i.e., the blow-down step in our cycle. However, we propose a different approach for the resolution, which allows us to include the process productivity in the model despite using equilibrium as well as to better represent real bed operation. This is enabled by the targeted use of neural networks, as explained in the following.

### Blow-Down Step

During the blow-down step, the total amount of gas leaving the column, *N*_out_, and the gas composition, *y*_*i*_, as well as the temperature, *T*, are calculated. The initial temperature and pressure are the same as that during adsorption, i.e., ambient conditions. When a vacuum is applied, a waste stream consisting mainly of the species present in the fluid phase is produced. Heating could also be applied to optimize the cycle, but given the difference in typical times of pressure and heat exchange, we neglect this and keep the model significantly simpler.

For the resolution of this step, the material and energy balances are solved for a discrete number of substeps, where the pressure is gradually decreased and gas is extracted from the bed until *p*_vac_ is reached. The pressure follows a time profile obtained from detailed 1D simulations, and it is temperature and material independent. Therefore, the material and energy balances for every step *k* of the blow-down are written as
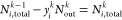
7
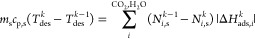
8where the superscript *k* refers to the current step of the blow-down and *k* – 1, to the preceding step. *N*_out_ is the total amount of gas that is removed from the column, and it is calculated for every step *k*. Since N_2_ is treated as an inert,  and therefore only considered as present in the fluid phase. As for the energy balance, *c*_p,s_ is the specific heat capacity of the sorbent; *T*_des_ is the desorption temperature, and Δ*H*_ads,*i*_ is the isosteric heat of adsorption for every species *i* adsorbing (i.e., H_2_O and CO_2_).  is calculated numerically from the energy balance for each substep *k* while Δ*H*_ads,*i*_ is computed for each substep *k* using the Clausius–Clapeyron expression, and, as suggested by Joss et al.,^32^ is approximated by including the temperature, pressure, and composition from the previous substep *k* – 1, i.e., . More details can be found in SI Section 1.6. The time length of the blow-down is directly retrieved from the vacuum pressure profile (see SI Section 1.1).

In previous works,^29,30,32^ the initial conditions of the blow-down step were set assuming that full bed saturation was reached during the adsorption step. However, full saturation is hardly achieved in fixed bed separations, as the front of the targeted species propagating in the bed is not perfectly sharp. Therefore, depending on the process specifications and characteristics, a certain level of undersaturation is always present in the bed, which affects the amount of targeted species that can be recovered. Here, we propose to overcome this intrinsic limitation of equilibrium models by computing the saturation level α via a neural network (NN) trained with rate-based simulations:
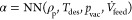
9In the following section, we provide more details about the neural networks. Accordingly, a level of saturation below 100% in the bed is assigned at the beginning of the blow-down, depending on the particle density, ρ_p_, the desorption temperature, *T*_des_, the vacuum pressure, *p*_vac_, and the volume feed stream, .

### Heating Step

During the heating step, the total amount of gas leaving the column, *N*_out_, and the gas composition, *y*_*i*_, as well as the external heat provided, *Q*_heating_, are calculated. The pressure is kept constant at *p*_vac_ during the whole step while the temperature is increased following a preassigned profile, which is calculated by fitting data from rate-based simulations. In contrast to the pressure profile, which is only dependent on the starting and end pressure, the temperature profile is dependent on the start and final temperature, the density and the heat capacity of the adsorbent material, and the bed pressure. Moreover, the length of the heating step is calculated via a dedicated neural network, whose training data are the same as that used for the saturation level. More details can be found in SI Section 1.2.

The material balance solved during heating is shown in eq 4, while the energy balance needs to be extended to include the external heating, *Q*_heating_:
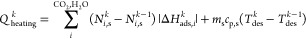
10

As for the blow-down, the differential equations of the isosteric heat of adsorption are presolved at each substep, *k*, using the pressure, temperature, and composition from the previous substep, *k* – 1 The initial conditions of the heating are the final conditions of the blow-down, and the heating step is calculated until the final desired temperature is reached.

### Cooling Step

During the cooling step, the exit valve is closed and the system is repressurized with ambient air and cooled by an external cooling. The initial conditions of the cooling step are equal to the final state of the heating step. The composition of the three components, *y*_*i*_, the air required for repressurization, *N*_in_, and the amount of cooling, and *Q*_cool_, are calculated. Similar to the previous steps, the repressurization and cooling proceeds incrementally by adding a small amount of air during each substep. The length of the cooling and the temperature/pressure profile are fixed according to the 1D model (more details can be found in SI Section 1.1). The cooling step is calculated until  reaches *T*_amb_, and the following balance equations are used
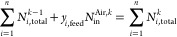
11
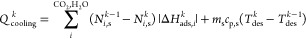
12

Besides using external cooling, it is also possible to implement open cooling with ambient air. In this case, in eq 12, the term  is substituted with . In addition, an outlet term needs to be added to the material balance in eq 11. The closed cooling system is generally the most efficient one, while for DAC applications, for simplicity reasons, open cooling may be preferred, which would lead to a lower recovery. For our further analysis, we therefore choose the closed cooling system.

### Adsorption Step

During this step, the total amounts of feed *N*_feed_ and waste *N*_waste_ are calculated. To keep the model simple and fast, the adsorption step is considered isothermal and isobaric at ambient temperature and pressure. The initial condition is set by the final condition of the cooling step, and the bed is then fed with ambient air until the CO_2_ saturation level set by the neural network is reached. Different from the previous steps, the adsorption is not divided into multiple *k* steps but solved for the final conditions directly. Because of the presence of H_2_O, CO_2_ can be either the weakly or the strongly adsorbed species depending on the material. Especially for cases with very low CO_2_ concentrations like in ambient air, CO_2_ is usually not the most strongly adsorbed species. Therefore, in order to avoid material balance errors, the adsorption step is divided into two substeps: the first substep is used to reach saturation of the strongly adsorbed species, while the second substep is used to reach the desired level of saturation of the weakly adsorbed species. Let us start with the case of CO_2_ as weakly adsorbed species. First, air is fed until the bed is saturated with H_2_O; here, both CO_2_ and H_2_O can adsorb. During the second substep, more air is fed until the bed reaches the CO_2_ saturation level fixed by the neural network, while H_2_O cannot adsorb anymore. On the other hand, if CO_2_ is the strongly adsorbed component, the adsorption step coincides with the first substep only, where the bed is fed until the CO_2_ saturation level from the NN is reached. The material balances solved during the first substep of adsorption are
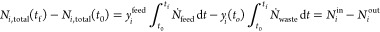
13where *N*_*i*,total_(*t*_0_) and *N*_*i*,total_(*t*_f_) are the total amount of component *i* in the column at the end of the cooling step and adsorption step, respectively;  is the concentration of the component in the feed stream; *y*_*i*_(*t*_o_) is the mole fraction of component *i* at the end of the cooling;  and  are the amount of species *i* fed and withdrawn form the column during the adsorption time, respectively. For the resolution of the material balances of the first substep of the adsorption and as far as H_2_O is the strongly adsorbed species,  corresponds to the conditions of full saturation in the column and eq 13:
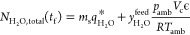
14If CO_2_ is the strongly adsorbed species, H_2_O in eq 14 is substituted with CO_2_. The material balances of the second substep of the adsorption are written as for the first substep (eq 13) but with the following differences:

- The initial conditions (*t*_0_) correspond to the end of the first adsorption substep.
corresponds to the saturation level assigned by the neural network.
- Water is treated as an inert; i.e., it flows through the column without adsorption.

The molar fraction of CO_2_ at the end of the second substep, , is equivalent to the initial composition of the blow-down step. Therefore, the total amount of air and the waste stream leaving the column are  and . The total time of the adsorption step can be determined by including the air velocity, *u*_air_, and the geometry of the considered sorbent (SI Section 1.4). Figure 2 gives an overview of the whole cycle with the input and output parameters for each step as well as the process performance parameters.

### Neural Networks

**Figure 3:**
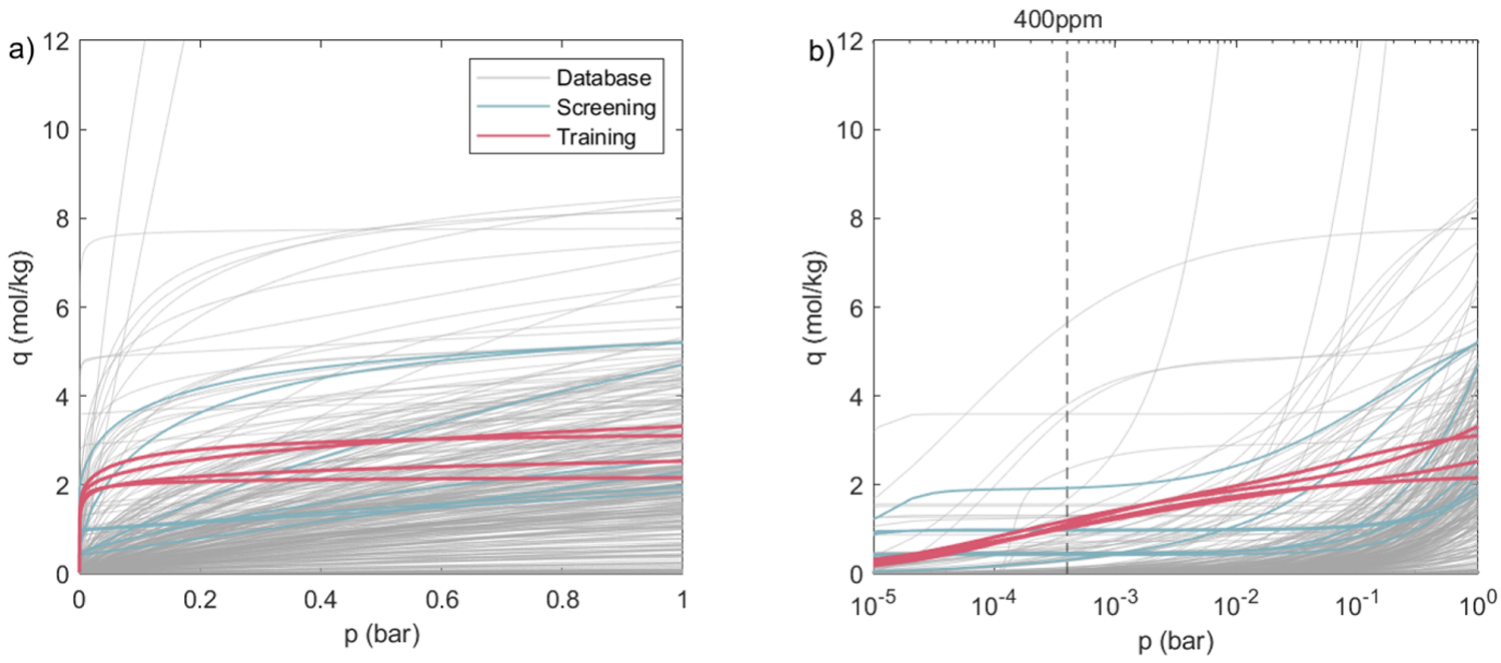
Overview over the isotherms included in the training data for the NN compared to the isotherms of the database: (a) linear scale and (b) logarithmic scale.

Two independent neural networks are used in the framework we propose, and they allow the inherent limitations of the equilibrium models to be overcome by providing: (i) the (under)saturation level of the bed α at the end of the adsorption step and (ii) the time required for heating, which in turns allows for an estimate of the productivity, an indicator typically computed in rate-based models exclusively (see the next subsection). This results in a more realistic and informative 0D model. The first NN directly provides α for assigned particle density, desorption temperature, vacuum pressure, and volume of gas fed (the variation of these cycle operating parameters corresponds to the exploration of different purity-recovery combinations). The second NN provides the time required to heat the bed to the regeneration temperature, *t*_heat_, which is typically the longest step in the cycle, for the assigned sorbent and particle density, sorbent specific heat, desorption temperature, and vacuum pressure. Indeed, the key to obtain meaningful neural networks is in the data set provided to the training step. Here, we used simulations carried out for eight different sorbent combinations obtained with the 1D rate-based model described in Sabatino et al. (the materials were taken from the same work).^37^ For the NN providing the heating time, a total of 4200 simulations were performed using the eight different material combinations and varying desorption temperature and vacuum pressure. For the NN providing the saturation level, we restricted the input data set to the optimal Pareto points of the eight sorbents described in Sabatino et al.,^37^ resulting in a total of 324 simulations. In Figure 3, we show the shape of the isotherms used for setting up the saturation neural network (red lines), those used for the database screening (gray), and those selected as optimum by the screening (light blue). We note that overall the isotherm shape is similar but that those used for the neural network training span a smaller area in the isotherm plane. The inclusion of additional sorbents in the 1D model data generation phase would likely strengthen the NN accuracy.

For both neural networks, the data sets were divided into training, validation, and testing data with a ratio of 60:20:20. The training was done using the Levenberg–Marquardt back-propagation method as implemented in the neural network toolbox provided in MATLAB. A summary of the input/output data for both neural networks is shown in Table 1.

**Table 1:** Summary of the Input, Output, and Boundary Conditions of the Neural Networks

input	output	# data sets	*T*_des_ range (K)	*p*_vac_ range (bar)
ρ_particle_, ρ_Material_, *c*_p,s_, *T*_des_, *p*_vac_	*t*_heat_	4200	363–400	0.1–0.8
ρ_particle_, *T*_des_, *p*_vac_, *V̇*_feed_	α	324	363–400	0.1–0.8

### Performance Indicators

The adsorption cycle can be evaluated via four performance indicators, which are calculated at the end of the simulation, i.e., the productivity , the specific thermal energy consumption , the CO_2_ recovery *r*, and the CO_2_ (dry) purity .
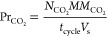
15
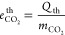
16
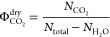
17
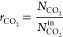
18where  is the amount of CO_2_ produced during the heating step, *t*_cycle_ is the total duration of the cycle, and *V*_s_ is the volume of the adsorbent. The time of the cycle required to compute the productivity is calculated by including all step times; i.e., *t*_cycle_ = *t*_BD_ + *t*_heat_ + *t*_cool_ + *t*_ads_. As shown in Figure 2, the time for the blow-down is obtained with a fitting function; the heating time is retrieved using a neural network; the cooling time is set to 350 s similar to our previous work;^37^ the time of the adsorption step is determined by the air velocity and the geometry of the sorbent as explained in the previous section.

The 0D model is implemented in MATLAB R2021, and the mass and energy balance equations are solved using the *lsqnonlin* solver and *trust-region-reflective* algorithm.^38^ The model takes less than 10 s to simulate one cycle using a laptop machine with an INTEL Core i7 2.50 GHz processor and 8.00 GB of RAM.

## Model Validation

The equilibrium model validation is carried out by comparing the results with a rate-based adsorption model. In the 1D model, the material and energy balances of a fixed-bed are typically expressed in differential form and are numerically solved in space and time until a cyclic steady state is reached. Moreover, the mass transfer is approximated with the linear driving force approach. The 1D model adopted for the validation has been used in multiple previous publications, and it has been shown to predict experimental results well.^39−42^ The detailed mathematical equations and the simulation parameters are reported in SI Section 1.8. Additional details can also be found in other previous publications.^27,31^

The validation of the 0D model is structured in two different steps. We need to recognize that not only does the 0D model have to reproduce fairly well the performance of a specific cycle simulated with the 1D model, but it also has to correctly identify the potential of a given material when the process is optimized. If both conditions hold, then the 0D model can be used to screen potential sorbents. Accordingly, first, we compare the profiles and performance indicators of a single simulation using the same input parameters (e.g., cycle times, inlet velocity, temperatures, and pressure). Second, we compare the results when the process is optimized; i.e., the input parameters are varied. More specifically, the optimization of the four-step VTSA process is carried out by minimizing the energy consumption and maximizing the productivity.^43^ The multiobjective optimization problem is formulated as follows:
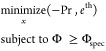
19where *x* is the decision variable, Φ is the purity, and Φ_spec_ is the required minimum purity. This constraint is imposed as a penalty *C* on the resulting objective function in the form of
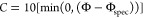
20

We would like to note that the minimum recovery Φ_spec_ value allowed in the optimization was set to 70%. This was required to include APDES-NFC, a DAC sorbent described in the early works of Climeworks’ founders, which we considered in previous work^37^ and in the 1D model simulations. This sorbent features a high porosity, and higher CO_2_ purity can only be reached when a preheating-to-waste step is included with the blow-down. However, the constraint is never active in the screening and for the validation of other sorbents (see next section and Figures S11, S12, S13). Therefore, the low-purity constraint does not affect the outcome of the fast screening.

The decision variables *x* for the 0D model are (i) the desorption temperature, (ii) the vacuum pressure, and (iii) the inlet feed velocity. The time of the different cycle steps depends on these parameters as well as on the material properties. The boundaries of the decision variables are given in Table S9. We repeat the same optimization for the 1D model using the same VTSA cycle and materials but adding the step times as variables.

The optimization of the 0D model is carried out using a particle swarm algorithm adapted for multiobjectives (MOPSOs), as implemented in MATLAB.^44^ The size of the particle and repository was set to 50 and the number of cycles, to 35. These parameters are smaller than the ones recommended by Coello et al.,^45^ but they did show the same accuracy. For the 1D model optimization, we follow the same approach reported in Sabatino et al.^37^ The results of the optimizations with the 0D and 1D models are compared in terms of the energy and productivity range for the eight materials discussed by Sabatino et al.^37^ The nomenclature of the materials can be found in Table S11 and includes four promising sorbents, namely, APDES-NFC,^46^ Tri-PE-MCM-41,^47^ MIL-101(Cr)-PEI-800,^48^ and Lewatit VP OC 1065,^49−51^ together with data for H_2_O isotherms of three different materials, i.e., APDES-NFC, Lewatit, and MCF-APS-hi.^52^ More details on the choice of these materials can be found in Sabatino et al.^37^

### Validation of a Specific Cycle Simulation

Here, we compare the 0D and 1D model for the same input parameters, i.e., same material, heating and cooling temperature, pressure, feed composition, feed velocity, and cycle times. The pure component isotherm equations as well as the parameters for the different materials are reported in SI Section 2. We carry out this validation for two different materials: case s2/E-A and Cr-MIL(101). Case s2/E-A was among the materials used to build the data collection adopted to develop the profile functions and the neural networks called in the 0D model. On the other hand, Cr-MIL(101) was not used for this purpose; thus, we use the Cr-MIL(101) validation to investigate the capability of the 0D model to predict the performance of the new materials. For all tested cases, we carry out the validation using air as feed, i.e., a direct air capture process.

The cycle times were defined by running the simulation with the 1D model and are long enough for the model to reach the vacuum pressure (*t*_BD_) and the desorption temperature (*t*_heating_) and to ensure a high saturation in the bed (*t*_ads_). The cooling time was set to *t*_cool_ = 350 s for all materials. This is done to ensure that the times as well as the final temperature and pressure are the same for both models. For this case, the 0D model structure was adapted to handle the times as additional input. Furthermore, the 1D model uses an equivalent temperature to include the enhancing effect of water on the CO_2_ adsorption; for consistency, this was also included in the 0D model for the validation. More details on this approach can be found in Sabatino et al.^37^ The process conditions for this validation are reported in Table 2.

**Table 2:** Process Conditions for the Validation of the 0D Model against the 1D Model

material	*T*_des_ (K)	*p*_vac_ (bar)	*V̇*_feed_(m^3^/s)	*t*_ads_ (s)	*t*_prod_ (s)	*t*_cool_ (s)	*t*_purge_ (s)	H_2_O isotherm
s2/E-A	373	0.27	8.90 × 10^–6^	3160	1500	350	30	APDES-NFC
Cr-MIL(101)	373	0.27	8.90 × 10^–6^	5000	4300	350	300	APDES-NFC

**Figure 4:**
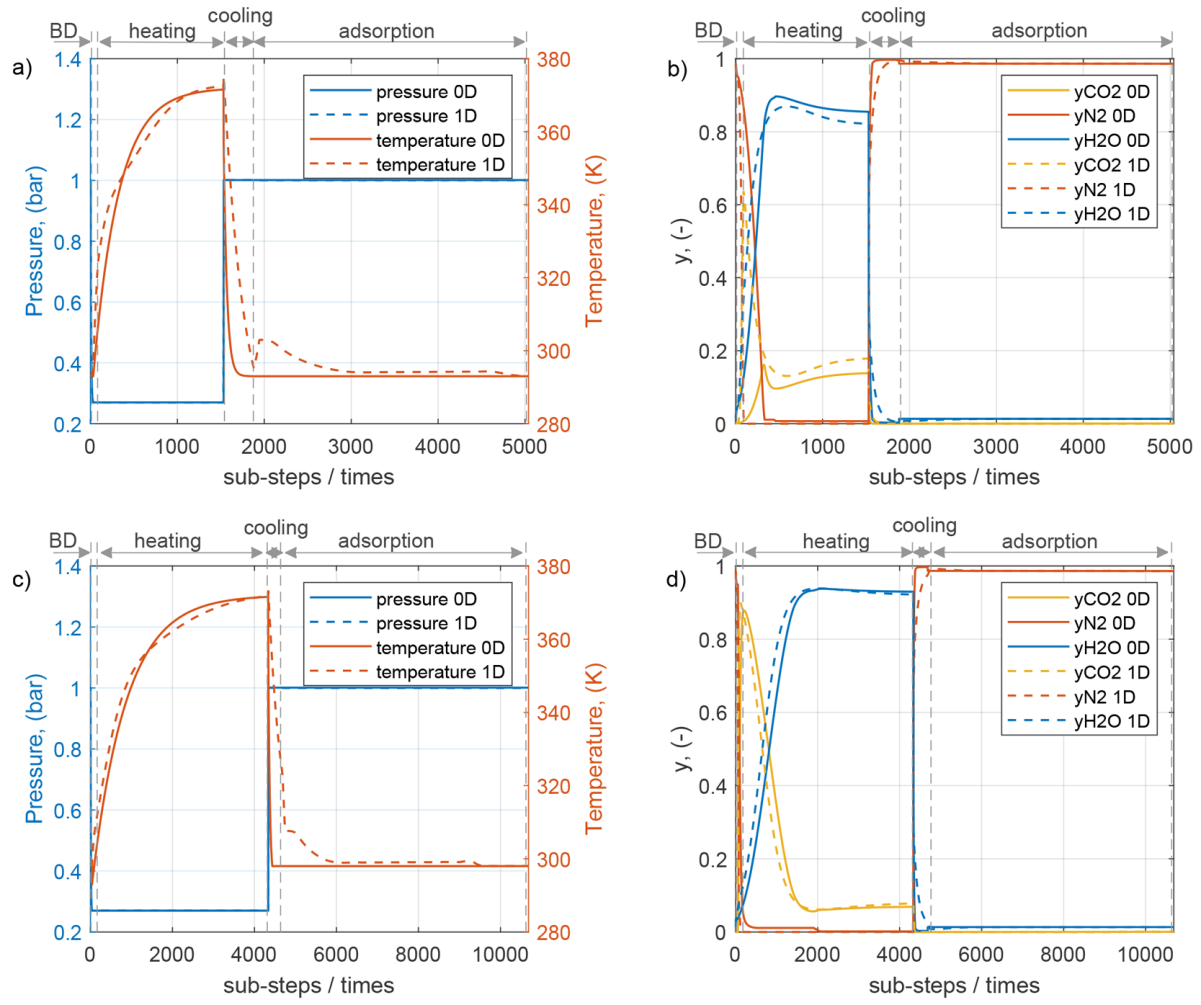
Temperature, pressure, and concentration profiles for the 0D and 1D models: (a, b) s1/E-A; (c, d) Cr-MIL(101). For the 0D model profiles, the adsorption step is plotted over time.

Figure 4 shows the temperature, pressure, and the molar fraction profiles of the three components for the 0D and 1D models. Figure 4a,b refers to the s2/E-A material, while Figure 4c,d refers to Cr-MIL(101). In addition, in Figure S5, the profiles of the adsorbed amounts of CO_2_ and H_2_O are added. Although the 0D model is significantly simpler than the detailed 1D model, the profiles are in good agreement. Notably, also, the concentration profiles show a good agreement between the models, which is hard to obtain for a well-stirred 0D equilibrium model. When looking at the main differences between the 0D and the 1D model, we notice the following. In the temperature profile (Figure 4a), a deviation is present for the adsorption step: for the 1D model, the temperature increases at the beginning of this step, while the 0D model shows a constant temperature. This is because we assume isothermal adsorption in the 0D model.

When looking at Figure 4c,d, i.e., the Cr-MIL(101) case, we notice that the model also predicts the profiles well when a new material is considered. This is an important feature of the model, and it confirms that the 0D model can effectively predict the performance of sorbents not used to build the NN functions and can therefore be used as a screening tool.

**Figure 5:**
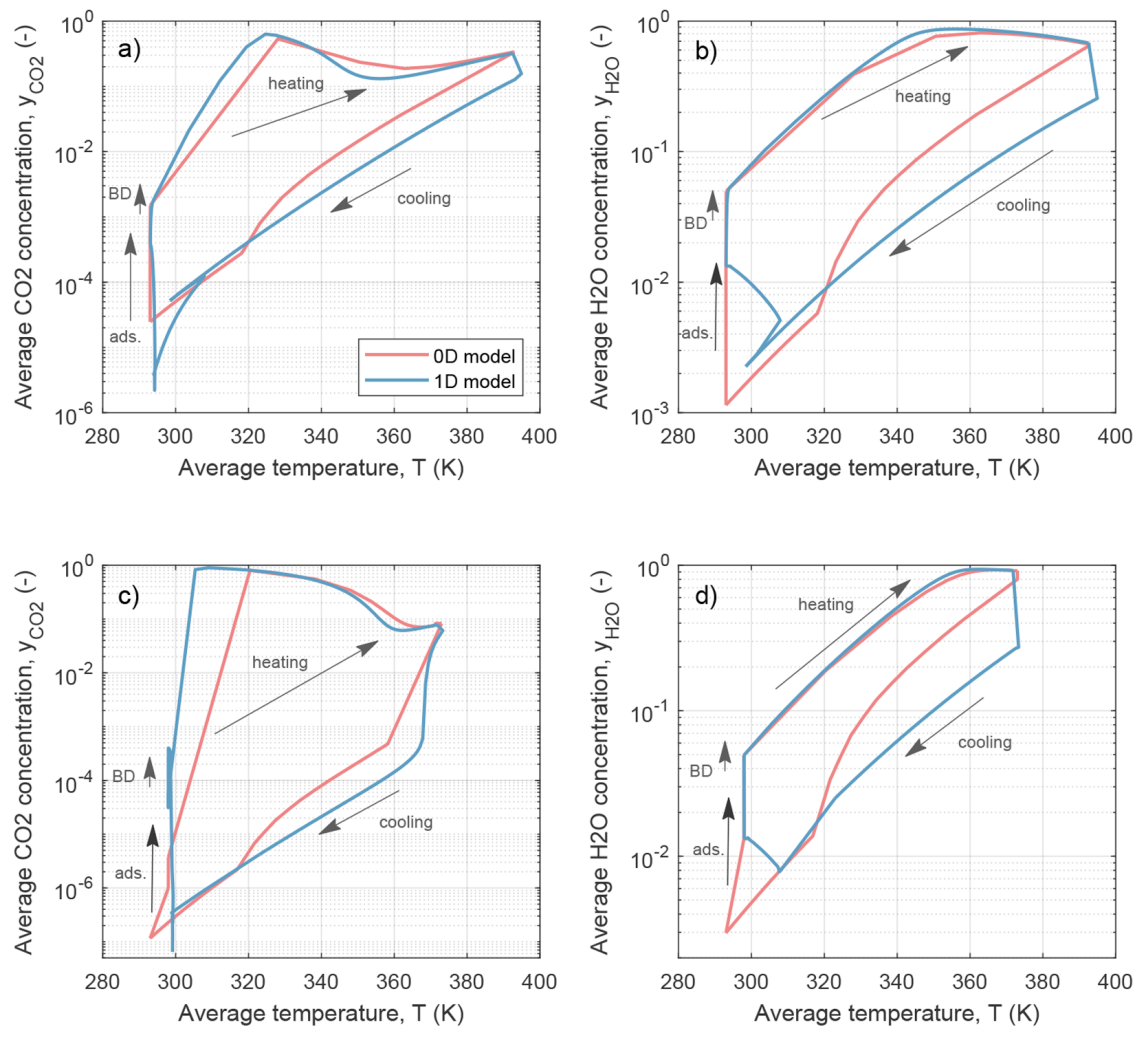
Composition profiles similar to Ajenifuja et al.^32^ of CO_2_ and H_2_O over the temperature for the case of s2/E-A (a, b) and Cr-MIL(101) (c, d). For the 1D model, the temperature and composition plots refer to average values over the bed at cyclic steady sate.

Figure 5 shows the molar fractions of CO_2_ and H_2_O as a function of the temperature during the cycle for both the 0D model and the 1D model. Also, here, Figure 5a,b shows the results for the sorbent case s2/A-E, while Figure 5c,d displays those for sorbent Cr-MIL(101) (the concentration–pressure profiles are reported in the SI). It should be noted that the composition profiles follow a similar shape for both sorbent s2/E-A and sorbent Cr-MIL(101).

**Figure 6:**
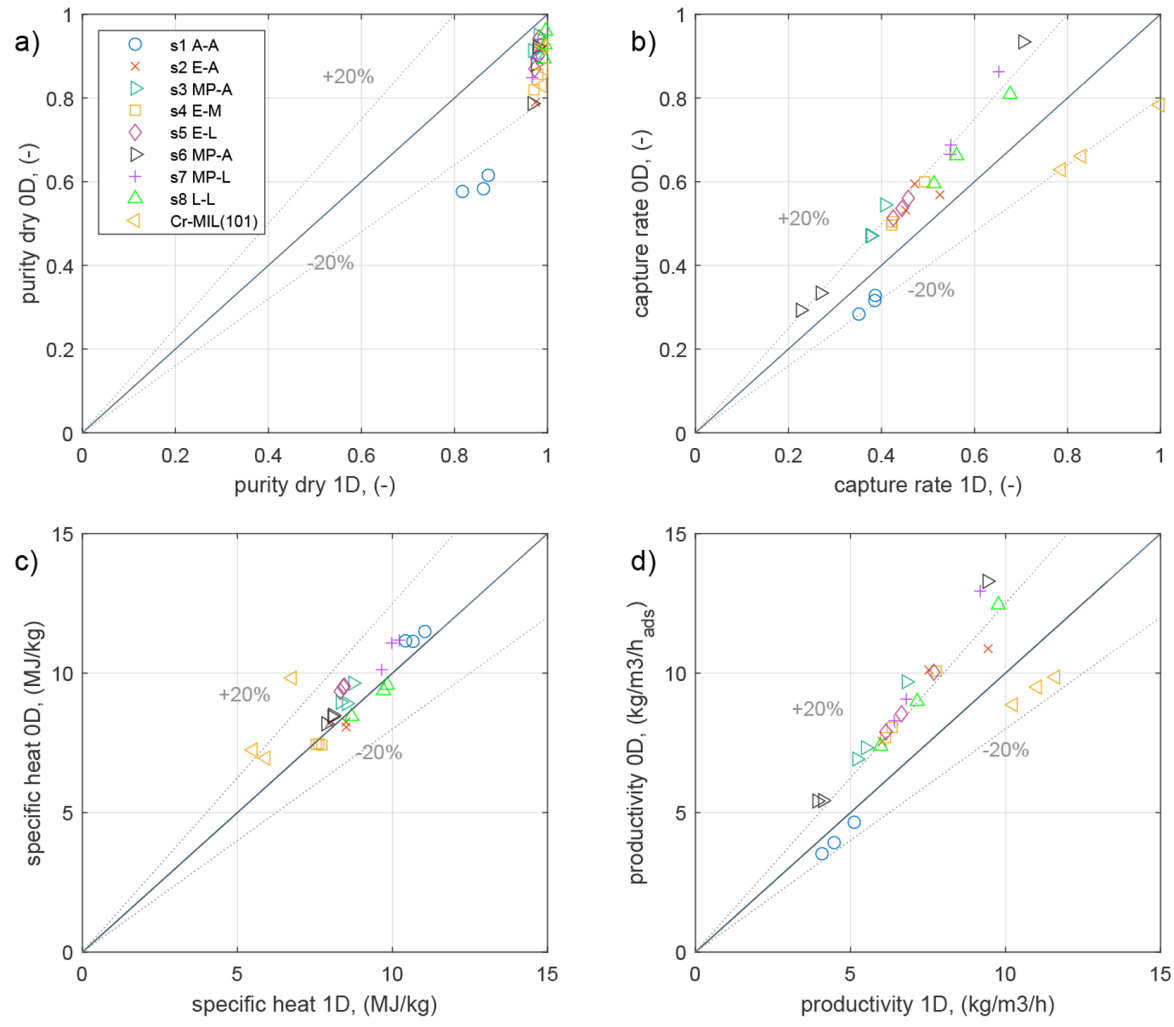
Parity plots of the performance parameters resulting from the 0D model and the 1D model. Each material is shown by a specific symbol and color, and each point features different input parameters for *T*_des_ and *p*_vac_. The dashed lines show a margin of 20%.

The overall performance of the 0D model with respect to the 1D model for all tested sorbents is shown in the parity plots in Figure 6, where the results are reported for the 9 different materials. Moreover, the parity plots for different CO_2_ concentrations in the feed and the same materials are reported in SI Section 2. The following can be concluded. (i) The purity predicted by the 0D model underestimates the 1D model, which is a consequence of the well-stirred approach. The results are however within a 20% gap with only material s1/A-A outside this gap. (ii) The capture rate is in good agreement and typically slightly overestimated by the 0D model (20% gap still applies). (iii) The specific thermal energy demand is in good agreement with the 1D simulation. (iv) The productivity is in fair agreement with the 1D model; this is particularly surprising, given that the 0D model is equilibrium based and the productivity is computed by means of a neural network function. The higher productivity stems from the higher capture rate of the 0D model. When considering the two additional cases for  and  vol., the parity plots show a similar agreement for energy and productivity and a better agreement for purity and capture rate (see SI Section 2.1).

Although the error for the predicted performance parameters is in the range of +/–20%, it must be noted that the 0D model does not aim to provide a very accurate prediction of the performance but rather at consistently predicting the performance of multiple sorbents. Therefore, to examine this feature better, we need to compare the capability of the model when process optimization is used.

### Validation of Sorbent Comparison and Optimization

As mentioned, the goal of the 0D model is to identify the most promising adsorbents from large databases of possible sorbents, where the ranking is done using technical key performance indicators. Therefore, we here benchmark the 0D model in terms of optimization of adsorbents. To this end, we consider again the 8 adsorbents used for the previous validation and compare the results of process optimization carried out using the 0D and the 1D models. The design variables considered with the 0D model are the desorption temperature, *T*_des_, the vacuum pressure, *p*_vac_, and the volume stream of the incoming air, . The same ranges across the different materials are considered for *T*_des_ and *p*_vac_, while for , the range is material-specific (the maximum air velocity is set by the minimum fluidization velocity). In contrast to the 0D model, the 1D model requires the cycle step times as input variables; in this case, the design variables and their upper and lower bounds are taken from Sabatino et al.^37^ and listed in Table S9.

**Figure 7:**
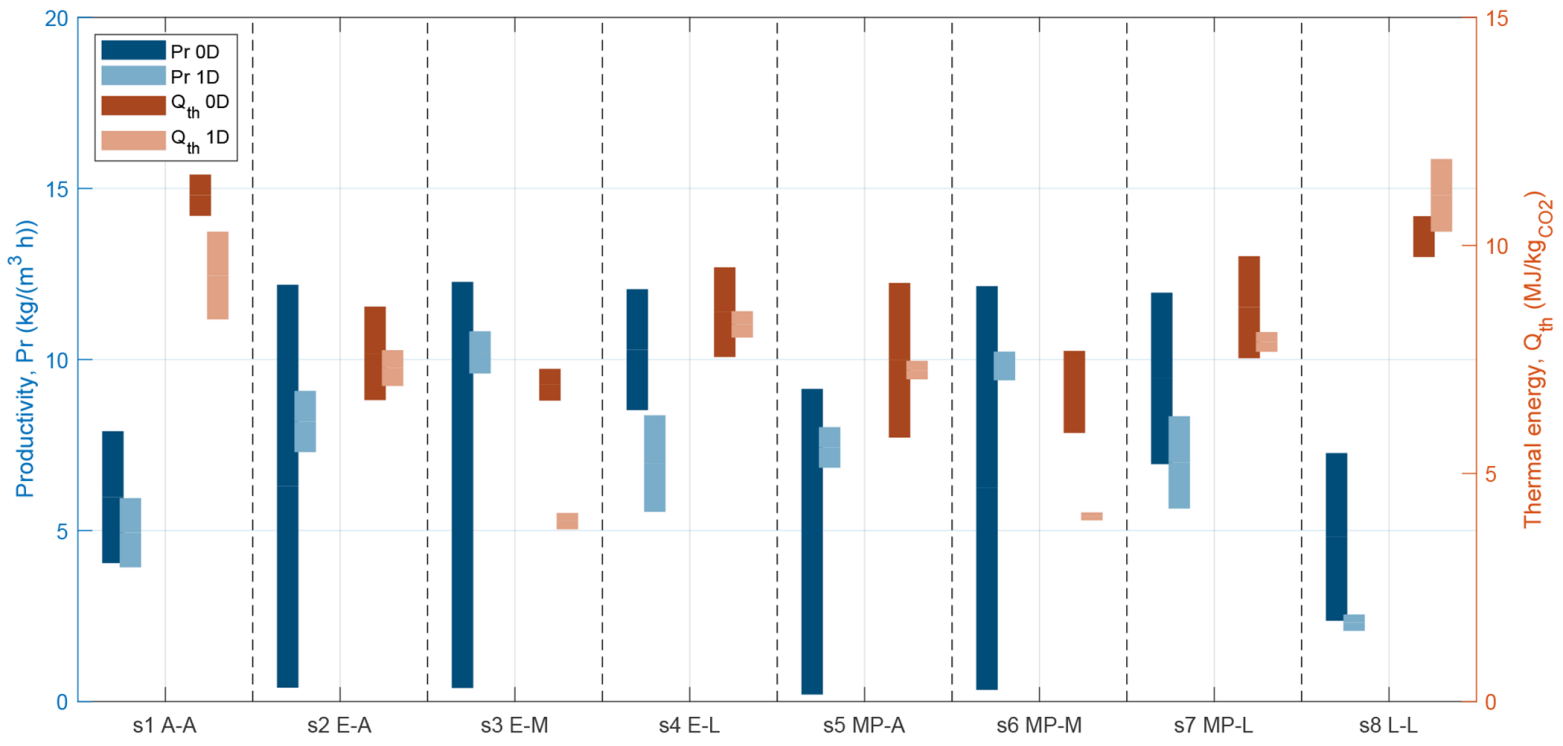
Resulting performance parameters (productivity in blue, thermal energy consumption in orange) from the 0D and 1D optimization. The nomenclature for the different sorbent cases can be found in Table S11.

As optimization results, Pareto curves with the optimal productivity-energy points are obtained. The detailed optimization results including purity, recovery, and decision variables are found in SI Section 2. To improve the visualization of the comparison between the models, the optimal Pareto points are depicted in Figure 7 as interval bars for both productivity (left *y* axis) and specific energy consumption (right *y* axis). As a comparison, the brighter bars show the corresponding results for the 1D model. When comparing the 0D and 1D models, we now aim to obtain a similar sorbent ranking (i.e., the most performing sorbents are identified) and similar range for the performance indicators.

Looking at the comparison, we first notice that the optimization results of the 0D model are in line with the 1D model; i.e., the simplified approach identifies similar values for energy consumption and productivity. Typically, the 0D model identifies broader ranges compared to the 1D model, especially for the productivity. The latter is however the most difficult indicator to extract from an equilibrium model. Second, we notice that the 0D model identifies the same well-performing and badly performing sorbents of the 1D (see Table 3 for a summary of the ranking). For the 0D model, the two best performing materials are s6/MP-A and s3/MP-A, while the two worst performing sorbents are s1/A-A and s8/L-L. The 1D model identifies the same worst sorbents and the same best sorbents (where the two best sorbents are swapped). These results let us conclude that the 0D model reliably reproduces the screening performance by the 1D model but in a fraction of the required time: around 2 h are needed for an optimization with the 0D model (per material) while from 8 h to several days are needed for the 1D model (per material). It should also be stressed that the 0D model shall not fully substitute the 1D model but complement it in the sorbent screening phase.

**Table 3:** Resulting Ranking of the Adsorbents for the Validation of the 0D Model Using the 1D Model^a^

0D model			1D model		
	Pr (kg/m^3^/h)	*Q*_th_ (MJ/kg)		Pr (kg/m^3^/h)	*Q*_th_ (MJ/kg)
s6 MP-M	12.1	7.7	s3 E-M	10.8	4.1
s3 E-M	+1%	–5%	s6 MP-M	–5%	0%
s2 E-A	0%	+13%	s2 E-A	–16%	+86%
s5 MP-A	–25%	+19%	s5 MP-A	+23%	+107%
s7 MP-L	–2%	+27%	s7 MP-L	–23%	+96%
s4 E-L	–1%	+24%	s4 E-L	–23%	+107%
s1 A-A	–35%	+50%	s1 A-A	–45%	+149%
s8 L-L	–40%	+38%	s8 L-L	–76%	+188%

a The values of the maximum productivity and the corresponding thermal energy consumption are given for the best performing adsorbent. For the remaining materials, the deviation to the best performing one is given in a percentage.

## Sorbents Screening

We applied the model described above to screen and rank a large number of possible sorbents. The screening was carried out by retrieving data from different sources, i.e., the NIST/ARPA-E database,^33^ adsorbents considered by Khurana and Farooq,^22^ and promising DAC sorbents from the literature not included in the previous sources.^36^ We do not limit the screening to specific classes of sorbents but consider, e.g., zeolites, activated carbon, and MOFs. Both real and hypothetical sorbents are included. The first objective is to demonstrate the potential of the 0D model by screening all the data mentioned above and by ranking the most promising adsorbents. The second objective is to identify the most promising sorbents for CO_2_ capture from diluted sources. To this end, we apply the screening to CO_2_ capture from air (400 ppm) and from sources at 0.1% vol CO_2_ and 1% vol CO_2_. These latter may be representative compositions found in stables and in the aluminum industry, respectively. Therefore, for the last two cases, an additional constraint is set, namely, the capture rate needs to be higher than 90%. For the DAC case on the other hand, the capture rate is not restricted.

### Screening Methodology

**Figure 8:**
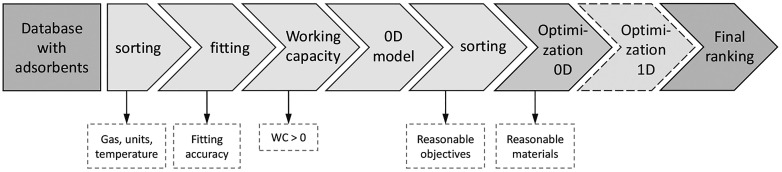
Overall screening approach.

The screening process includes several steps, which are shown in Figure 8. All screening tools are made available as open source online; see the SI. As a first step, the isotherm data of the NIST/ARPA-E needs to be retrieved from the online database and preliminary filtered to exclude adsorbents that cannot be further considered. This includes, e.g., isotherm availability for the gas of interest or converting the units of the data. More details are provided in Figure S10. In the next step, isotherm fitting is carried out for the remaining adsorbents. Since the isotherm of the adsorbents can take various shapes, we allow for automatic selection among three common isotherm models during the fitting, i.e., the Langmuir–Freundlich, the Toth-cp, and the s-shaped methods (these three isotherm models can capture a wide range of experimental isotherm shapes). The fitting approach is further described in SI Section 3.

In the next step, the working capacity is calculated to identify those materials with positive capacity for the CO_2_ adsorption of interest. A summary of these conditions is reported in Table 4. While these process conditions are fixed here, the screening could be carried out for varying inputs. Thereafter, the 0D model is run for all materials with a positive working capacity. When no H_2_O isotherm is provided in the database, we include the H_2_O isotherm of APDES-NFC^46^ and Lewatit^49^ with a fitting provided by Sabatino et al.^37^ (the H_2_O uptake of the APDES-NFC isotherm lies somewhere in the middle, while Lewatit adsorbs higher amounts of H_2_O). Moreover, we consider the case of no water adsorption with a dry feed. For the screening process, no enhancing effect of water on the CO_2_ adsorption was considered, as no specific and reliable information/data is generally available.

**Table 4:** Boundary Conditions for the Material Screening

	*T*_des_ (K)	*T*_ads_ (K)	*p*_vac_ (bar)	*p*_ads_ (bar)	*y*_CO2_^feed^ (%)	*y*_H2O_^feed^ (%)	*y*_N2_^feed^ (%)
DAC (400 ppm)	373	293	0.1	1.001	0.04	1.34	98.62
0.1%	373	293	0.1	1.001	0.1	1.34	98.56
1.0%	373	293	0.1	1.001	1.0	1.34	97.66

Another issue present for most of the materials, especially those from the NIST/ARPA-E database, concerns the availability of physical property data (and associated units), like the material and particle density and heat capacity. For the cases where one or more properties are not available, the following generic assumptions are made: ρ_material_ = 1130 kg/m^3^, particle void fraction (ϵ_particle_) = 0.35, and *c*_p,s_ = 1070 J/kg/K.^22,32^ While this is certainly a simplification, any other assumptions would result in a similar outcome.

In the next step, the results of the 0D model are sorted: materials with specific energy consumption higher than 100 MJ/kg_CO_2__ are excluded from further consideration. For the remaining adsorbents, an optimization is carried out using the 0D model. The upper and lower bounds of the decision variables are the same as for the validation of the exemplary isotherm mentioned in the previous section (see Table S9). The optimization results allow for a final ranking of the sorbents. Possibly, the most promising sorbents are further evaluated by optimization with the 1D model.

### Screening Results

**Figure 9:**
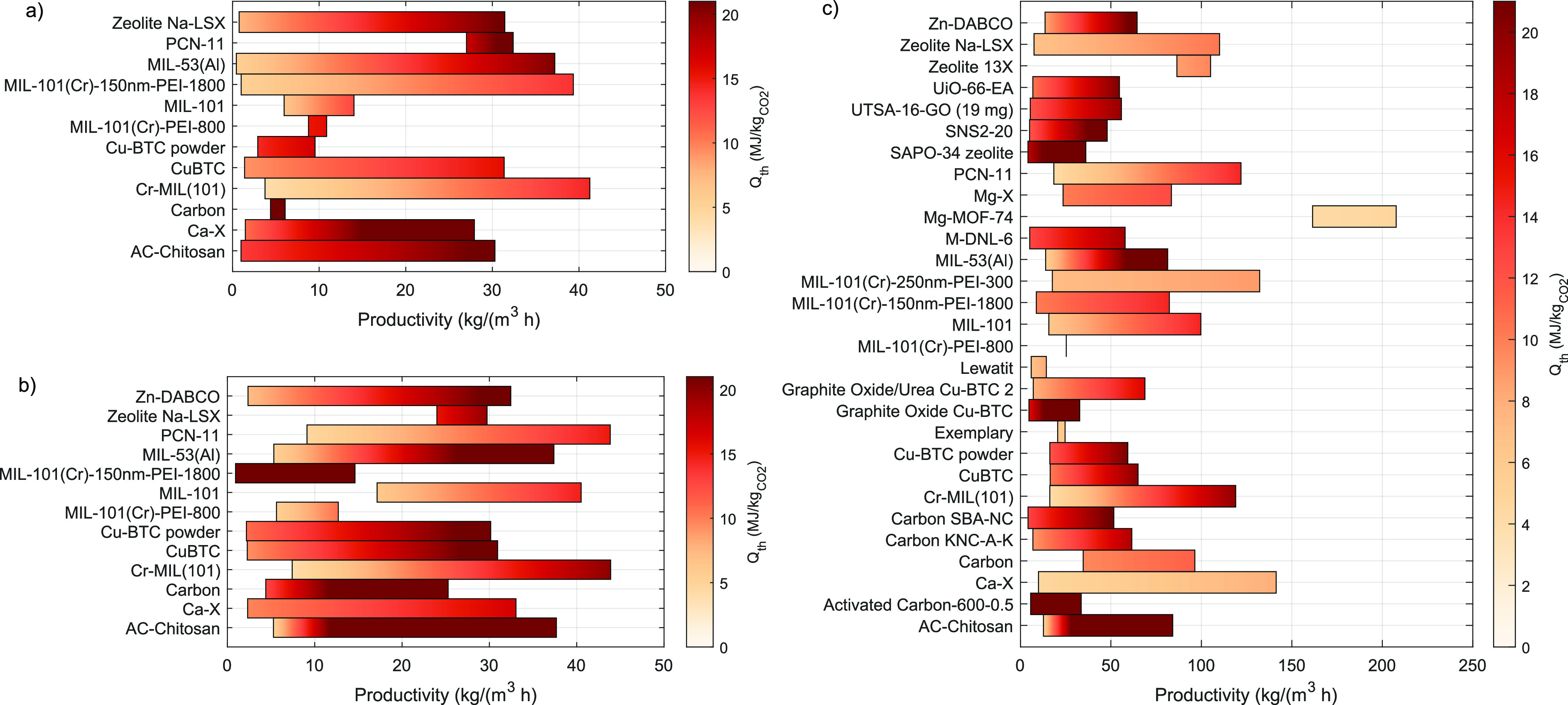
Resulting objectives for three case studies: (a) humid air feed stream with , (b) , and (c) . The best performing adsorbents show a high productivity Pr, given on the *x* axis, and a low specific thermal energy demand *Q*_th_ represented by the color bar. The upper limit of the color bar is set to a specific value to make the differences and best performing materials visual. The actual energy consumption can therefore be higher than the limit shown by the bar.

The screening considers initially around 2500 different materials for which nearly 8000 isotherms are fitted and sorted. For the DAC case, only 12 materials show a positive working capacity and reasonable performance parameters. 13 and 30 materials were found for  and , respectively. Here, we would like to remind the reader that for the two latter cases the capture rate is constrained to be higher than 90% while it can vary freely for the DAC case. The screening results are shown in Figure 9, while Table 5 reports the ranking of the 10 best performing adsorbents for the three different cases. The ranking is based on the minimum specific energy consumption and the maximum productivity. Figure 9 shows that, as expected, the specific energy consumption is decreasing and the productivity is increasing for higher CO_2_ concentrations in the feed. The model consistently predicts that, for an increase in productivity, more thermal energy is needed. Notably, a few particular sorbents can be identified for all applications.

**Table 5:** Resulting Rankings Showing the 10 Best Performing Materials for Three Different Cases with Varying CO_2_ Concentrations in the Feed and Including Water^a^

	400 ppm	0.1%	1.0%
1	Cr-MIL(101) (2)	PCN-11	Mg-MOF-74
2	MIL-101 (1)	MIL-101	Ca-X
3	CuBTC (3)	Cr-MIL(101)	MIL-101(Cr)-250 nm-PEI-300
4	MIL-53(Al) (4)	Ca-X	zeolite Na-LSX
5	Zn-DABCO (5)	zeolite Na-LSX	zeolite 13X
6	MIL-101(Cr)-PEI-800 (6)	Cu-BTC powder	PCN-11
7	Lewatit (8)	CuBTC	MIL-101
8	exemplary (7)	MIL-53(Al)	carbon^b^
9	zeolite Na-LSX (10)	Zn-DABCO	Cr-MIL(101)
10	Ca-X (9)	MIL-101(Cr)-PEI-800	Mg-X

a The names of the sorbents correspond to the naming in the NIST database. The ranking results for 400 ppm (second column) using the 1D model are added next to the materials in brackets. The deviation of the productivity and thermal energy consumption between the best perfoming material and the rest is given in Tables S13–S15.

b Activated carbon.

For DAC, the MOFs Cr-MIL(101) and MIL-101 have the best performance in terms of both productivity, which can reach 20 kg/(m^3^h), and energy; the latter can be potentially as low as 4.1 MJ_th_/kg_CO_2__. For 0.1%, PCN-11 is also an interesting sorbent in addition to the MOFs for DAC. The maximum productivity increases to above 40 kg/(m^3^h). Finally, for the 1.0% case, the MOFs Mg-MOF-74, MIL-101(Cr)-250 nm-PEI-399, and Ca-X are identified as the most promising with energy consumption as low as 4.3 MJ_th_/kg_CO_2__ and maximum productivity above 100 kg/(m^3^h). Nicely, zeolite 13-X is also identified as one of the most performing sorbents, in line with what has been reported for postcombustion CO_2_ capture with VSA and TSA cycles.

All materials short-listed from screening the 0.1% case are included in the results of the 1.0% case. The materials of the 0.04% case, on the other hand, are not all included in the higher CO_2_ concentration cases, since for the DAC case we do not constrain the capture rate: the capture rate of the three excluded materials is lower than 90% (see Figures S11 and S12).

For the DAC case, an optimization with the 1D model is also carried out for the short-listed sorbents, and the corresponding material ranking is reported in brackets in Table 5. The rankings for the two models is again very similar, and the two best adsorbents are consistently identified. The three cases using a different water isotherm, i.e., from the Lewatit sorbent, are very similar to the screening with the APDES-NFC isotherm, and the resulting ranking is the same (see the SI). When looking at the screening cases using a dry feed stream, the results, on the contrary, are very different. This was expected since the concentration profiles are very different.

## Discussion

In this work, we showed that equilibrium models can effectively contribute to the overall design of the adsorption processes for CO_2_ capture from diluted sources and from air. Especially, they are useful tools to map the preliminary performance regions and to identify promising sorbents from large databases. Coupling equilibrium models with machine learning further enhances the outcome, e.g., by providing good estimates of the process productivity. However, as for all models, there are a few limits that are worth stressing (which are particularly relevant for scientists interested in reusing our MATLAB package provided on GITHUB).

First, it should be kept in mind that the purpose of the 0D model is not to provide accurate predictions, for that, rate-based models shall be used, but to enable (i) otherwise nonviable simulations, e.g., high-throughput materials screening, and (ii) and a better understanding of the process performance in an early stage of development. The 0D model should be consistent with the rate-based models’ predictions but should not aim at substituting them.

Second, it is important to include in the 0D model the adsorption of all relevant species and adopt suitable adsorption models. For example, in this work, we have considered N_2_ as an inert, which is acceptable as far as N_2_ adsorption is negligible (e.g., in amine-functionalized sorbents), but which should be included when dealing with more traditional sorbents (e.g., zeolite 13X). N_2_ must also be included when extending this model framework to nondiluted CO_2_ capture applications, e.g., NGCC, coal, and industrial sources. Along a similar line, in this work, we have neglected H_2_O competition and enhancement effects: this should be corrected as soon as more experimental data become available.

The last limitation we would like to discuss here concerns the neural network modules. As all data-driven models, the quality of the NNs depends on the quality of the data input. In the development of the NN used in our model, we considered a limited number of materials (see Figure 3) and kinetic data, and we used such NNs for extrapolation to different isotherms. While this led to outcomes in line with the rate-based model, the performance of the NN could be improved by adding training data from additional materials (e.g., considering the finding of this work as shown in Figure 9). When more experimental data become available, most of all about kinetics, the model should be updated accordingly: first, by retraining the NNs, second by rethinking the overall model structure. One possibility along the latter line might include the use of AI as surrogate model for equilibrium and using the kinetics indicators to drive the sorbent selection (see 1).

## Conclusions

In this paper, we presented a new equilibrium-based 0D model for the rapid simulation of vacuum temperature swing adsorption cycles. The model, which builds upon the key assumption of well-mixed conditions in the bed, was developed to enable a fast, yet reliable screening of sorbents for CO_2_ removal from diluted sources, e.g., direct air capture applications. Nonetheless, the formulation is generic and portable to other separations of interest as far as the model is adapted to grasp the key separation characteristics. To this end, we extended the approaches presented in the literature for equilibrium-based adsorption models by embedding neural network submodels trained from rate-based simulations, by including H_2_O in the feed (i.e., CO_2_ is not necessarily the strongly adsorbed species), and by considering the vacuum temperature adsorption cycle. The resulting model can predict the separation performance (capture rate and purity), the specific energy consumption, and the productivity. The latter is enabled thanks to the embedding of machine learning, as equilibrium models do not provide rate-connected performance. The resulting 0D model can simulate a VTSA cycle in less than 10 s and a full cycle optimization in less than 2 h, therefore significantly lowering the computing time, especially on standard desktops and thus enabling a large screening of new materials.

We have shown that the resulting 0D model can predict fairly well the different performance indicators of VTSA cycles. To this end, we compared the model with the results of a more sophisticated 1D rate-based model. The validation included the comparison of specific fixed cycles for several materials in terms of performance indicators and temperature/composition profiles and also the comparison of the outcome of cycle optimizations for different sorbents. The findings confirm that (i) the 0D model reproduces well specific cycles and (ii) returns similar metrics when optimizing cycles; i.e., it is capable of substituting more sophisticated models in the large screening of materials.

Finally, we applied the 0D model to the screening of several thousands of sorbents, which were obtained from the NIST/ARPA-E database and additional literature.^22,33,36,37^ We carried out the screening to assess CO_2_ capture from air and from other diluted sources . The sorbent screening also included additional steps that are required to retrieve and polish the source data. We identified 12, 13, and 28 promising materials for the DAC, the , and the cases, respectively. In all cases, a couple of sorbents stood out as particularly promising in terms of both energy consumption and productivity. As final comparison, we run the optimization of the DAC promising sorbents with the 1D model; the outcome results were fully consistent with the 0D model.

Overall, we can conclude that equilibrium models, and particularly the one we propose here, are a powerful tool for sorbent screening that could reliably substitute more sophisticated models. We showed that this also holds true when using more complicated cycles, i.e., VTSA, and when considering more challenging separations, i.e., from (ultra)diluted sources.

## Supporting Information Available

Details on the modeling approach; fitting parameters for profiles and times; list of equations for 0D model; list of equations for 1D model; tables containing modeling parameters; parity plots showing validation results for additional cases; table with isotherm model equations; flowchart showing screening and fitting approach; figures with purity and recovery for screening results; figures with screening results for additional cases; tables listing resulting materials from screening with DOIs (PDF)

## Notes

The following files are available free of charge and provided in the GitHub repository at https://github.com/UU-ER/SorbentsScreening0D: 0D model, MATLAB code of the 0D model; Screening NIST, MATLAB code to extract and screen the materials of the NIST/ARPA-E Database.

## Footnotes

1 We would like to acknowledge that the idea of swapping the use of AI from surrogate for kinetics to surrogate for equilibrium was suggested by one of the reviewers of this work.
