# Supplementary material for: A Machine Learning-Aided Equilibrium Model of VTSA Processes for Sorbents Screening Applied to CO2 Capture from Diluted Sources
Source: Ind Eng Chem Res. 2022 Sep 6;61(37):14004–19. doi: 10.1021/acs.iecr.2c01695 (PMC9501812; doi:10.1021/acs.iecr.2c01695)
Supplement: Supplementary file 1 — ie2c01695_si_001.pdf [file ie2c01695_si_001.pdf]

Supplementary Materials for

A machine learning-aided equilibrium model of VTSA processes for sorbents screening applied to CO_2 capture from diluted sources

Alexa Grimm[a] and Matteo Gazzani[a,b,⋆]

[a] Universiteit Utrecht, Copernicus Institute of Sustainable Development, Princetonlaan 8a, 3584 CB, Utrecht, The Netherlands

[b] Sustainable Process Engineering, Chemical Engineering and Chemistry, Eindhoven University of Technology, 5612 AP Eindhoven, The Netherlands

⋆Corresponding author. Email: m.gazzani@uu.nl

1 Modelling details

1.1 Blow-down step: pressure profile and time

The time for the blow-down step is mainly dependent on the vacuum pressure. By fitting simulation data from the 1D model, the following equation was received

$$t_{\text{BD}} = \frac{-10p_{\text{vac}} + a}{b} \tag{S1}$$

with the parameters listed in Table S1. Since the profile mainly varies with pressure and does not show a notable dependence on the density or temperature of the material, the fitting was carried out for a small dataset for one material (case s2 E-A) and different vacuum pressures over time.

Table S1: Fitting parameters for the time of the blow-down step.

a	b
20.70	20 000

The pressure at each sub-step p_{vac}^k is calculated using the following equation

$$p_{\text{vac}}^k = \frac{1}{10}\left(\frac{a}{p_{\text{vac}}^b} exp\left(ct_{\text{BD}}^k\right) + p_{\text{vac}} exp\left(\frac{d}{p_{\text{vac}}^b} t_{\text{BD}}^k\right)\right) \tag{S2}$$

The fitted parameters are listed in Table S2

Table S2: Fitting parameters for the pressure profile of the blow-down step.

a	b	c	d
0.18627	0.85	-0.1238	-7.138 e^{-05}

1.2 Heating step: temperature profile and time

The training data of the neural network for determining the heating time can be found in Figure S1.

The temperature profile was received by fitting several profiles retrieved from simulations using the 1D model. The profile is dependent on the desorption temperature and the heating time

$$T_{\text{heat}}^k = (a + T_{\text{des}} + b \cdot T_{\text{des}}^c) * atan\left(\frac{t_{\text{heat}}^k - d \cdot 10^9 \cdot T_{\text{des}}^e}{f}\right) + (T_{\text{des}} - g) \cdot h \tag{S3}$$

The fitted parameters are listed in Table S3.

Table S3: Fitting parameters for the temperature profile of the heating step.

a	b	c	d	e	f	g	h
-687.31	2791.97	0.346	-661.53	-4.296	42.005	-34162.55	0.00867

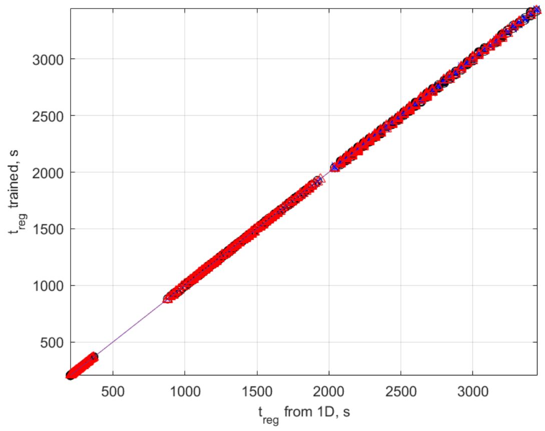

Figure S1: Resulting training data (blue symbols) from the neural network together with the validation data (red) and the testing data (black).

1.3 Cooling step: profile

The time of the cooling step is fixed to 350 seconds, similar to the 1D model simulations. The temperature profile is calculated similar to the heating step, by fitting data from the 1D model. The fitting equation is

$$T^k_{\text{cooling}} = 293 + \left(aT_{\text{des}} exp\left(b \cdot t^k_{\text{cool}}\right)\right) \tag{S4}$$

with the parameters listed in Table S4.

Table S4: Fitting parameters for the temperature profile of the cooling step.

a	b
0.17485	-0.02629

1.4 Adsorption step

The total time of the adsorption step can be determined by including the air velocity u_{air} and the geometry of the considered sorbent

$$t_{\text{ads}} = \frac{N^{\text{feed}}_{\text{ads}} M_{\text{air}}}{u_{\text{air}} A \rho_{\text{air}}} \tag{S5}$$

with M_{air} being the molar mass of the air, A the column cross section and ρ_{air} the density of the air.

1.5 Saturation level

Since we are considering an equilibrium model, the saturation at the end of the adsorption step or rather at the begin of the blow-down step is generally 100%. A more realistic picture of the saturation level gives the simulation with the detailed model, which shows a lower saturation level. Figure S2 shows the saturation level calculated for the Pareto points from the optimization of three different materials (the same optimization as used for the validation). The saturation level α was calculated for each grid point of the bed by calculating the actual loading referred to the full saturation at ambient conditions

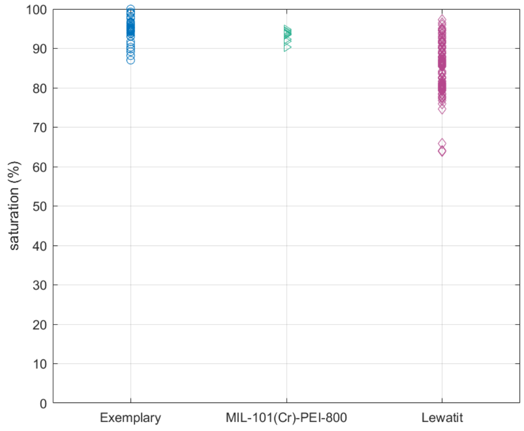

Figure S2: Saturation level from simulations using the 1D model.

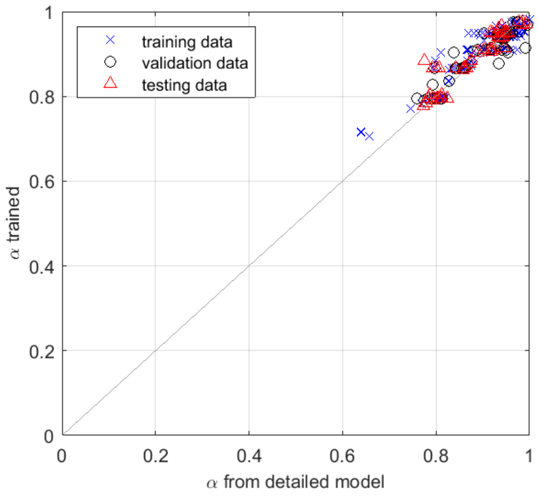

Figure S3: Training, testing and validation results.

$$\alpha = \sum_{n=1}^{N_{\text{grid}}} \frac{q^*_{CO_2}\left((T_{\text{amb}}, p_{\text{amb}}, y_{CO_2,\text{feed}}\right) - q^n_{CO_2}\left((T, p, y_{CO_2}\right)}{q^*_{CO_2}\left((T_{\text{amb}}, p_{\text{amb}}, y_{CO_2,\text{feed}}\right)} \tag{S6}$$

with $q^n_{CO_2}$ being the actual loading at the respective grid point and $q^*_{CO_2}$ the loading at equilibrium.

Using this data, a neural network was trained to determine the saturation level dependent on the particle density ρ_p, the desorption temperature T_{des}, the vacuum pressure p_{vac} as well as the air volume flow $\dot{V}_{\text{feed}}$

$$\alpha = NN(\rho_{\text{particle}}, T_{\text{des}}, p_{\text{vac}}, \dot{V}_{\text{feed}}) \tag{S7}$$

The training, testing and validation results can be found in Figure S3.

1.6 Isosteric heat of adsorption

The isosteric heat of adsorption (ΔH_{ads}) represents the strength of the adsorbate-adsorbent interaction and is defined as the difference between the activation energy for adsorption and desorption. It can be calculated from the Clausius-Clapeyron [1] equation

$$\left(\frac{\partial(\ln p_{\mathrm{CO2}})}{\partial T}\right)_{q_{\mathrm{equ}}} = \frac{-\Delta H_{\mathrm{ads}}}{RT^2} \tag{S8}$$

where p_{CO2} is the partial pressure of CO_2 (Pa), T is the absolute temperature (K) and R is the universal gas constant.

1.7 Equations 0D model

Table S5: Equations 0D model (first part).

Blow-down step

Known: $q^0_{\mathrm{CO_2}}, q^0_{\mathrm{H_2O}}, y^0_{\mathrm{CO_2}}, y^0_{\mathrm{H_2O}}, y^0_{\mathrm{N_2}}, p$

Calculated: $N_{\text{out}}, y^k_{\mathrm{CO_2}}, y^k_{\mathrm{H_2O}}, y^k_{\mathrm{N_2}}, T^k$

$$\left(m_s q^{k-1}_{\mathrm{CO_2}} + \frac{y^{k-1}_{\mathrm{CO_2}} p^{k-1} V\epsilon}{RT^{k-1}}\right) - y^k_{\mathrm{CO_2}} N^k_{\text{out}} - \left(m_s q^k_{\mathrm{CO_2}} + \frac{y^k_{\mathrm{CO_2}} p^k V\epsilon}{RT^k}\right) = 0 \tag{S9}$$

$$\left(m_s q^{k-1}_{\mathrm{H_2O}} + \frac{y^{k-1}_{\mathrm{H_2O}} p^{k-1} V\epsilon}{RT^{k-1}}\right) - y^k_{\mathrm{H_2O}} N^k_{\text{out}} - \left(m_s q^k_{\mathrm{H_2O}} + \frac{y^k_{\mathrm{H_2O}} p^k V\epsilon}{RT^k}\right) = 0 \tag{S10}$$

$$\left[\left(m_s q^{k-1}_{\mathrm{CO_2}} + \frac{y^{k-1}_{\mathrm{CO_2}} p^{k-1} V\epsilon}{RT^{k-1}}\right) + \left(m_s q^{k-1}_{\mathrm{H_2O}} + \frac{y^{k-1}_{\mathrm{H_2O}} p^{k-1} V\epsilon}{RT^{k-1}}\right) + \left(\frac{y^{k-1}_{\mathrm{N_2}} p^{k-1} V\epsilon}{RT^{k-1}}\right)\right] - N^k_{\text{out}} - \left[\left(m_s q^k_{\mathrm{CO_2}} + \frac{y^k_{\mathrm{CO_2}} p^k V\epsilon}{RT}^k\right) + \left(m_s q^k_{\mathrm{H_2O}} + \frac{y^k_{\mathrm{H_2O}} p^k V\epsilon}{RT^k}\right) + \left(\frac{y^k_{\mathrm{N_2}} p^k V\epsilon}{RT^k}\right)\right] = 0 \tag{S11}$$

$$m_s c_p \left(T^k - T^{k-1}\right) - \Delta H_i s, CO2^{k-1} \left(m_s q^{k-1}_{\mathrm{CO_2}} - m_s q^k_{\mathrm{CO_2}}\right) - \Delta H_i s, H2O^{k-1} \left(m_s q^{k-1}_{\mathrm{H_2O}} - m_s q^k_{\mathrm{H_2O}}\right) = 0 \tag{S12}$$

$$1 - y^k_{\mathrm{CO_2}} - y^k_{\mathrm{H_2O}} - y^k_{\mathrm{N_2}} = 0 \tag{S13}$$

Heating step

Known: $q^0_{\mathrm{CO_2}}, q^0_{\mathrm{H_2O}}, y^0_{\mathrm{CO_2}}, y^0_{\mathrm{H_2O}}, y^0_{\mathrm{N_2}}, p, T$

Calculated: $N_{\text{out}}, y^k_{\mathrm{CO_2}}, y^k_{\mathrm{H_2O}}, y^k_{\mathrm{N_2}}, Q_{\text{ex}}$

$$\left(m_s q^{k-1}_{\mathrm{CO_2}} + \frac{y^{k-1}_{\mathrm{CO_2}} p^{k-1} V\epsilon}{RT^{k-1}}\right) - y^k_{\mathrm{CO_2}} N^k_{\text{out}} - \left(m_s q^k_{\mathrm{CO_2}} + \frac{y^k_{\mathrm{CO_2}} p^k V\epsilon}{RT^k}\right) = 0 \tag{S14}$$

$$\left(m_s q^{k-1}_{\mathrm{H_2O}} + \frac{y^{k-1}_{\mathrm{H_2O}} p^{k-1} V\epsilon}{RT^{k-1}}\right) - y^k_{\mathrm{H_2O}} N^k_{\text{out}} - \left(m_s q^k_{\mathrm{H_2O}} + \frac{y^k_{\mathrm{H_2O}} p^k V\epsilon}{RT^k}\right) = 0 \tag{S15}$$

$$\left[\left(m_s q^{k-1}_{\mathrm{CO_2}} + \frac{y^{k-1}_{\mathrm{CO_2}} p^{k-1} V\epsilon}{RT^{k-1}}\right) + \left(m_s q^{k-1}_{\mathrm{H_2O}} + \frac{y^{k-1}_{\mathrm{H_2O}} p^{k-1} V\epsilon}{RT^{k-1}}\right) + \left(\frac{y^{k-1}_{\mathrm{N_2}} p^{k-1} V\epsilon}{RT^{k-1}}\right)\right] - N^k_{\text{out}} - \left[\left(m_s q^k_{\mathrm{CO_2}} + \frac{y^k_{\mathrm{CO_2}} p^k V\epsilon}{RT}^k\right) + \left(m_s q^k_{\mathrm{H_2O}} + \frac{y^k_{\mathrm{H_2O}} p^k V\epsilon}{RT^k}\right) + \left(\frac{y^k_{\mathrm{N_2}} p^k V\epsilon}{RT^k}\right)\right] = 0 \tag{S16}$$

$$1 - y^k_{\mathrm{CO_2}} - y^k_{\mathrm{H_2O}} - y^k_{\mathrm{N_2}} = 0 \tag{S17}$$

$$Q^k_{\text{ex}} - m_s c_p \left(T^k - T^{k-1}\right) + \Delta H_i s, CO2^{k-1} \left(m_s q^{k-1}_{\mathrm{CO_2}} - m_s q^k_{\mathrm{CO_2}}\right) + \Delta H_i s, H2O^{k-1} \left(m_s q^{k-1}_{\mathrm{H_2O}} - m_s q^k_{\mathrm{H_2O}}\right) = 0 \tag{S18}$$

Table S6: Equations 0D model (second part).

Cooling step

Known: $q^0_{\mathrm{CO_2}}, q^0_{\mathrm{H_2O}}, y^0_{\mathrm{CO_2}}, y^0_{\mathrm{H_2O}}, y^0_{\mathrm{N_2}}, p, T$

Calculated: $N_{\mathrm{in}}, y^k_{\mathrm{CO_2}}, y^k_{\mathrm{H_2O}}, y^k_{\mathrm{N_2}}, Q_{\mathrm{cool}}$

$$\left(m_s q^{k-1}_{\mathrm{CO_2}} + \frac{y^{k-1}_{\mathrm{CO_2}} p^{k-1} V\epsilon}{RT^{k-1}}\right) + y^k_{\mathrm{CO_2}} N^k_{\mathrm{in}} - \left(m_s q^k_{\mathrm{CO_2}} + \frac{y^k_{\mathrm{CO_2}} p^k V\epsilon}{RT^k}\right) = 0 \tag{S19}$$

$$\left(m_s q^{k-1}_{\mathrm{H_2O}} + \frac{y^{k-1}_{\mathrm{H_2O}} p^{k-1} V\epsilon}{RT^{k-1}}\right) + y^k_{\mathrm{H_2O}} N^k_{\mathrm{in}} - \left(m_s q^k_{\mathrm{H_2O}} + \frac{y^k_{\mathrm{H_2O}} p^k V\epsilon}{RT^k}\right) = 0 \tag{S20}$$

$$\left[\left(m_s q^{k-1}_{\mathrm{CO_2}} + \frac{y^{k-1}_{\mathrm{CO_2}} p^{k-1} V\epsilon}{RT^{k-1}}\right) + \left(m_s q^{k-1}_{\mathrm{H_2O}} + \frac{y^{k-1}_{\mathrm{H_2O}} p^{k-1} V\epsilon}{RT^{k-1}}\right) + \left(\frac{y^{k-1}_{\mathrm{N_2}} p^{k-1} V\epsilon}{RT^{k-1}}\right)\right] + N^k_{\mathrm{in}} - \left[\left(m_s q^k_{\mathrm{CO_2}} + \frac{y^k_{\mathrm{CO_2}} p^k V\epsilon}{RT}^k\right) + \left(m_s q^k_{\mathrm{H_2O}} + \frac{y^k_{\mathrm{H_2O}} p^k V\epsilon}{RT^k}\right) + \left(\frac{y^k_{\mathrm{N_2}} p^k V\epsilon}{RT^k}\right)\right] = 0 \tag{S21}$$

$$1 - y^k_{\mathrm{CO_2}} - y^k_{\mathrm{H_2O}} - y^k_{\mathrm{N_2}} = 0 \tag{S22}$$

$$Q^k_{\mathrm{cool}} - m_s c_p \left(T^k - T^{k-1}\right) + \Delta H_i s, CO2^{k-1}\left(m_s q^k_{\mathrm{CO_2}} - m_s q^{k-1}_{\mathrm{CO_2}}\right) + \Delta H_i s, H2O^{k-1}\left(m_s q^k_{\mathrm{H_2O}} - m_s q^{k-1}_{\mathrm{H_2O}}\right) = 0 \tag{S23}$$

Adsorption step

Sub-step 1

Known: $q^{\mathrm{cool}}_{\mathrm{CO_2}}, q^{\mathrm{cool}}_{\mathrm{H_2O}}, y^{\mathrm{cool}}_{\mathrm{CO_2}}, y^{\mathrm{sat}}_{\mathrm{CO_2}}, p, T$

Calculated: $N_{\mathrm{waste}}, N_{\mathrm{feed}}, y_{\mathrm{H_2O}}, y_{\mathrm{N_2}}$

$$\left(m_s q^{\mathrm{cool}}_{\mathrm{CO_2}} + \frac{y^{\mathrm{cool}}_{\mathrm{CO_2}} p_{\mathrm{amb}} V\epsilon}{RT_{\mathrm{amb}}}\right) + y^{\mathrm{feed}}_{\mathrm{CO_2}} N_{\mathrm{feed}} - \left(m_s q^{\mathrm{sat}}_{\mathrm{CO_2}} + \frac{y^{\mathrm{sat}}_{\mathrm{CO_2}} p_{\mathrm{amb}} V\epsilon}{RT_{\mathrm{amb}}}\right) - y^{\mathrm{cool}}_{\mathrm{CO_2}} N_{\mathrm{waste}} = 0 \tag{S24}$$

$$\left(m_s q^{\mathrm{cool}}_{\mathrm{H_2O}} + \frac{y^{\mathrm{cool}}_{\mathrm{H_2O}} p_{\mathrm{amb}} V\epsilon}{RT_{\mathrm{amb}}}\right) + y^{\mathrm{feed}}_{\mathrm{H_2O}} N_{\mathrm{feed}} - \left(m_s q_{\mathrm{H_2O}} + \frac{y_{\mathrm{H_2O}} p_{\mathrm{amb}} V\epsilon}{RT_{\mathrm{amb}}}\right) - y^{\mathrm{cool}}_{\mathrm{H_2O}} N_{\mathrm{waste}} = 0 \tag{S25}$$

$$\frac{y^{\mathrm{cool}}_{\mathrm{N_2}} p_{\mathrm{amb}} V\epsilon}{RT_{\mathrm{amb}}} + y^{\mathrm{feed}}_{\mathrm{N_2}} N_{\mathrm{feed}} - \frac{y_{\mathrm{N_2}} p_{\mathrm{amb}} V\epsilon}{RT_{\mathrm{amb}}} - y^{\mathrm{cool}}_{\mathrm{N_2}} N_{\mathrm{waste}} = 0 \tag{S26}$$

$$1 - y^{\mathrm{sat}}_{\mathrm{CO_2}} - y_{\mathrm{H_2O}} - y_{\mathrm{N_2}} = 0 \tag{S27}$$

Table S7: Equations 0D model (third part).

Sub-step 2

Known: $q^1_{\mathrm{CO_2}}, y^{\mathrm{sat}}_{\mathrm{CO_2}}, y^{\mathrm{sat}}_{\mathrm{H_2O}}, y^1_{\mathrm{N_2}}, p, T$

Calculated: $N_{\mathrm{waste}}, N_{\mathrm{feed}}, y^2_{\mathrm{N_2}}$

$$\left(m_s q^1_{\mathrm{CO_2}} + \frac{y^1_{\mathrm{CO_2}} p_{\mathrm{amb}} V \epsilon}{R T_{\mathrm{amb}}}\right) + y^{\mathrm{feed}}_{\mathrm{CO_2}} N_{\mathrm{feed}} - \left(m_s q^{\mathrm{sat}}_{\mathrm{CO_2}} + \frac{y^{\mathrm{sat}}_{\mathrm{CO_2}} p_{\mathrm{amb}} V \epsilon}{R T_{\mathrm{amb}}}\right) - y^1_{\mathrm{CO_2}} N_{\mathrm{waste}} = 0 \qquad \text{(S28)}$$

$$\frac{y^1_{\mathrm{N_2}} p_{\mathrm{amb}} V \epsilon}{R T_{\mathrm{amb}}} + y^{\mathrm{feed}}_{\mathrm{N_2}} N_{\mathrm{feed}} - \frac{y^2_{\mathrm{N_2}} p_{\mathrm{amb}} V \epsilon}{R T_{\mathrm{amb}}} - y^1_{\mathrm{N_2}} N_{\mathrm{waste}} = 0 \qquad \text{(S29)}$$

$$1 - y^{\mathrm{sat}}_{\mathrm{CO_2}} - y^{\mathrm{sat}}_{\mathrm{H_2O}} - y^1_{\mathrm{N_2}} = 0 \qquad \text{(S30)}$$

1.8 Details 1D model

Table S8: Equations for 1D adsorption model [2]

Total mass balance
$$\epsilon_t \frac{\partial \left(\frac{p}{T}\right)}{\partial t} + \frac{\partial \left(\frac{up}{T}\right)}{\partial x} + \rho_b R \sum_{j=1}^{N} \frac{\partial n_j}{\partial t} = 0 \quad \text{(S31)}$$
Component mass balance
$$\epsilon \frac{\partial \left(\frac{y_i p}{T}\right)}{\partial t} + \frac{\left(\frac{u y_i p}{T}\right)}{\partial x} + \rho_b R \frac{\partial n_i}{\partial t} = 0 \quad i = 1, ..., N \quad \text{(S32)}$$
Mass transfer (Linear driving force model)
$$\frac{\partial n_i}{\partial t} = k_i \left(n_i^{eq} - n_i\right) \quad i = 1, ..., N \quad \text{(S33)}$$
Energy balance for the fixed bed
$$\left(\epsilon_t C_g + \rho_b C_s + \rho_b C_{\text{ads}}\right) \frac{\partial p}{\partial t} - \epsilon \frac{\partial p}{\partial t} + u C_g \frac{\partial T}{\partial x} - \rho_b \sum_{j=1}^{N} \left(-\Delta H_j\right) \frac{\partial n_j}{\partial t} + \frac{4 h_{\text{wall-bed}}}{d_i} \left(T - T_W\right) = 0 \quad \text{(S34)}$$
Energy balance for the bed wall
$$\frac{\partial T_W}{\partial t} = \frac{4}{C_{\text{p,W}} \left(d_0^2 - d_i^2\right)} \left(d_i h_{\text{wall-bed}} \left(T - T_W\right) + d_0 h_{\text{fluid-wall}} \left(T_{\text{fluid}} - T_w\right)\right) \quad \text{(S35)}$$
Momentum balance (Ergun equation)
$$\frac{\partial p}{\partial x} = -\frac{150 \mu \left(1 - \epsilon_b\right)^2}{\epsilon_b^3 d_p^2} u - \frac{1.75 \left(1 - \epsilon_b\right) \rho}{\epsilon_b^3 d_p} \|u\| u \quad \text{(S36)}$$

The decision variables for the detailed process simulation are listed in Table S9.

Table S9: Design variables for optimizing the process, including lower and upper bound. The upper bound for the air volume stream is calculated using the minimum fluidization velocity [3]. For the 0D model only T_{des}, p_{vac} and $\dot{V}_{air}$ are considered as decision variables.

Variable	Range			
	APDES-NFC	MIL-101(Cr)-PEI-800	Lewatit VP OC 106	Exemplary
t_{ads} (s)	100-10000			
t_{des} (s)	50-2200			
t_{purge} (s)	1-400			
p_{vac} (bar)	0.1-0.9			
T_{des} (K)	363-373			
ΔT_{purge} (K)	1-60			
$\dot{V}_{air}$ (m^3/s)	$2.0 \cdot 10^{-7}$ $-1.2 \cdot 10^{-5}$	$2.0 \cdot 10^{-7}$ $-8.9 \cdot 10^{-6}$	$2.0 \cdot 10^{-7}$ $-3.7 \cdot 10^{-6}$	$2.0 \cdot 10^{-7}$ $-8.9 \cdot 10^{-6}$

Details regarding the modeling of the solid sorbent process are shown in Table S10.

Table S10: Parameters for modeling the adsorption unit using the 1D model.

Parameter	Value	Source
Plate containing sorbent		
Length	0.05 m	
Internal radius	0.005 m	
External radius	0.005001 m	
Heat capacity wall	2.457×10^6 J/(K m^3)	
Heat transfer fluid/wall/bed	6.7 W/(m^2K)	Modeled using [4]
Contactor geometry		
LxWxH	1.5x1.5x1.5m	[4]
Void fraction, $\epsilon = 1 - V_{sorbent}/V_{aircontator}$	0.6	[4]

2 Validation

Table S11: Different combinations for CO_2 and H_2O isotherms.

CO_2 isotherm	H_2O isotherm		
	APDES-NFC	MCF-APS-hi	Lewatit VP OC 106
APDES-NFC	s1:**A-A**	-	-
Exemplary	s2: **E-A**	s3: **E-M**	s4: **E-L**
MIL-101(Cr)-PEI-800	s5: **MP-A**	s6: **MP-M**	s7: **MP-L**
Lewatit VP OC 106	-	-	s8: **L-L**

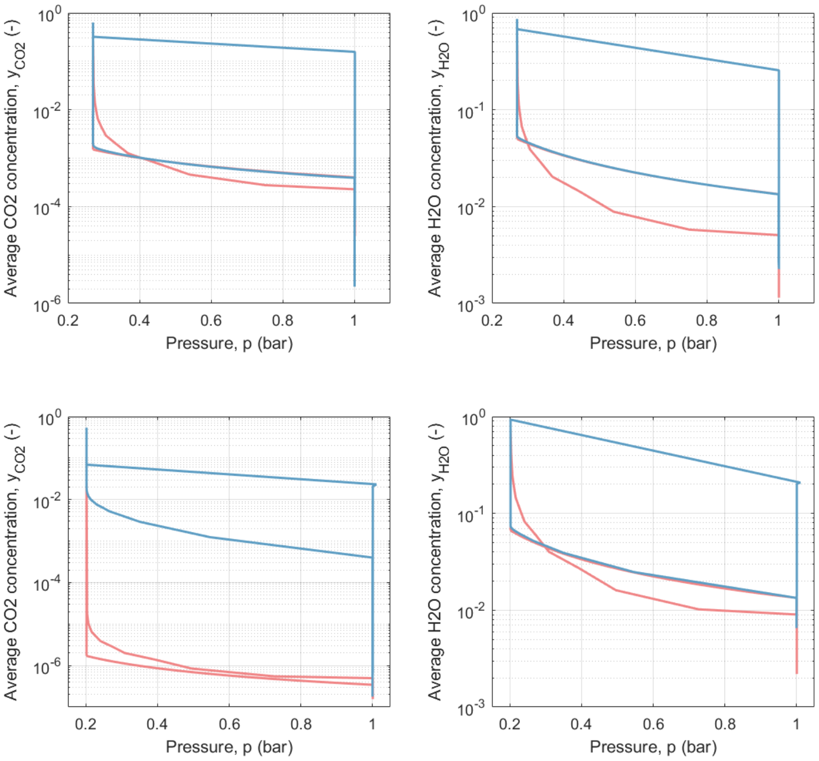

Figure S4: Composition of CO_2 and H_2O over the pressure for the whole cycle for case s2/E-A (a) and b)), and Cr-MIL(101) (c) and d)).

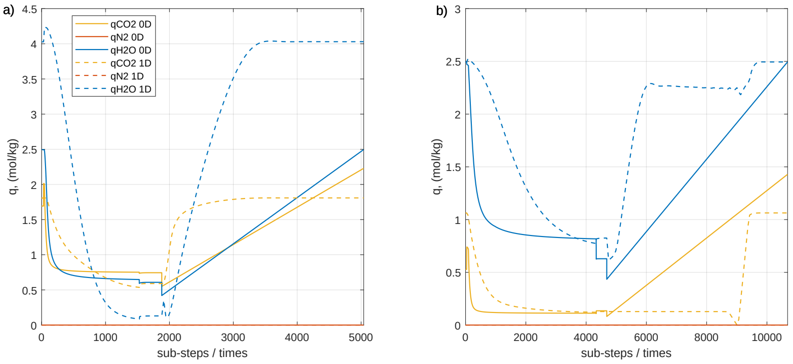

Figure S5: Profiles of the adsorbed amount during one cycle, corresponding to Figure 4 in the main text. a) shows case s2/E-A and b) Cr-MIL(101).

2.1 Parity plots varying CO_2 concentration

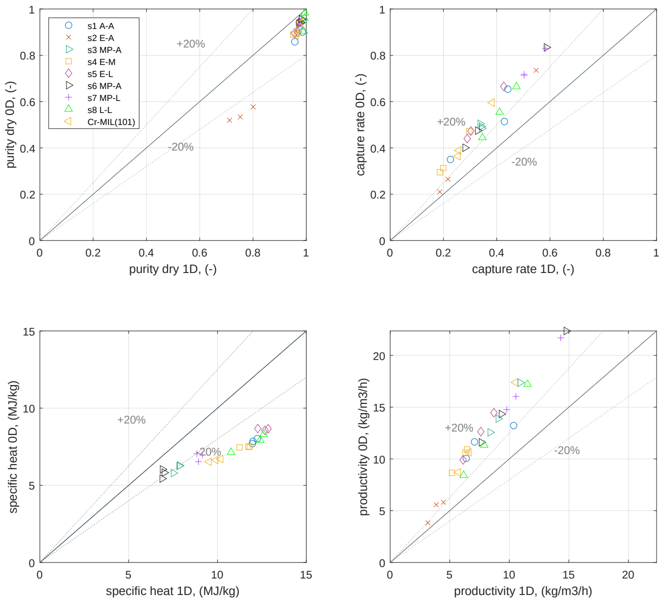

Figure S6: Parity plot for $y_{CO_2} = 0.1\%$.

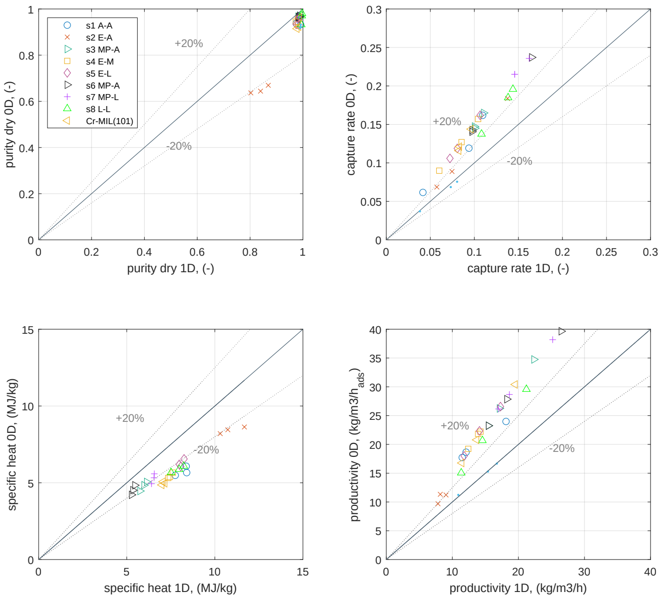

Figure S7: Parity plot for $y_{CO_2} = 1.0\%$.

The range of the resulting purity and recovery for both the 0D and the 1D model are shown in Figure S8.

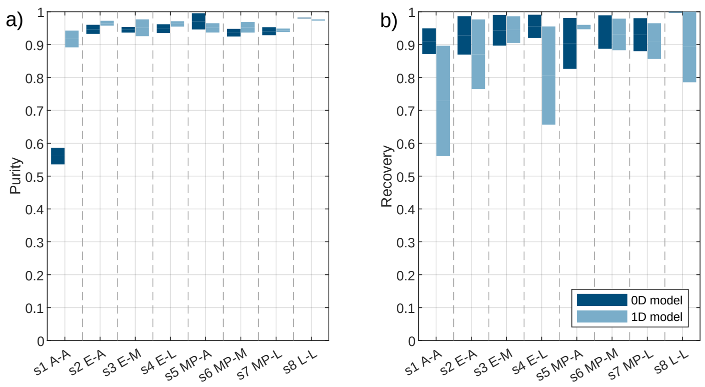

Figure S8: a) Purity and b) capture rate from optimizing the materials during the validation for the 0D and 1D model.

3 Screening

Table S12: Equations of the different isotherm models.

Model		Equation
Toth-cp	equ. 1	$q_{\mathrm{CO_2}} = n_{\mathrm{s,c}} \frac{b_c p y_{\mathrm{CO_2}}}{\left(1+\left(b_c p y_{\mathrm{CO_2}}\right)^{t_c}\right)^{1/t_c}} + n_{s,p} \frac{b_p p y_{\mathrm{CO_2}}}{\left(1+\left(b_p p y_{\mathrm{CO_2}}\right)^{t_p}\right)^{1/t_p}}$
		$n_{s,c/p} = n_{s0,c/p} \exp\left(Xi_{c/p}\left(1-\frac{T}{T_0}\right)\right)$
		$b_{c/p} = b_{0,c/p} \exp\left(\frac{\Delta H_{c/p}}{RT_0}\right)\left(\frac{T_0}{T}-1\right)$, $t_{c/p} = t_{0,c/p} + \alpha_{c/p}\left(1-\frac{T_0}{T}\right)$
DSL	equ. 2	$q_{\mathrm{CO_2}} = n_1 \frac{b\left(y_{\mathrm{CO_2}} p\right)}{1+b\left(y_{\mathrm{CO_2}} p\right)} + n_2 \frac{d\left(y_{\mathrm{CO_2}} p\right)}{\left(1+d\left(y_{\mathrm{CO_2}} p\right)\right)}$
		$b = b_0 exp\left(\frac{H_b}{RT}\right)$, $d = d_0 exp\left(\frac{(H_d}{RT}\right)$
Toth	equ. 3	$q_{\mathrm{CO_2}} = n_s \frac{b p y_{\mathrm{CO_2}}}{\left(1+\left(b p y_{\mathrm{CO_2}}\right)^{t}\right)^{1/t}}$
		$n_s = n_{s0} \exp\left(Xi\left(1-\frac{T}{T_0}\right)\right)$
		$b = b_0 \exp\left(\frac{\Delta H}{RT_0}\left(\frac{T_0}{T}-1\right)\right)$, $t = t_0 + \alpha\left(1-\frac{T_0}{T}\right)$
Langmuir-Freundlich	equ. 4	$q_{\mathrm{CO_2}} = n_s \frac{\left(b p y_{\mathrm{CO_2}}\right)^{1/t}}{\left(1+\left(b p y_{\mathrm{CO_2}}\right)^{1/t}\right)}$
		$n_s = n_{s0} exp\left(Xi\left(1-\frac{T}{T_0}\right)\right)$
		$b = b0 * exp\left(\frac{\Delta H}{(RT_0)}\left(\frac{T_0}{T}-1\right)\right)$
		$t = t_0 + \alpha\left(1-\frac{T_0}{T}\right)$
S-shaped	equ. 5	$q_{\mathrm{CO_2}} = q_L(1-w) + q_U w$
		$q_L = \frac{q_{L,0} b_L\left(y_{\mathrm{CO_2}} p\right)}{\left(1+b_L\left(y_{\mathrm{CO_2}} p\right)\right)}$
		$b_{L/U/H} = b_{L/U/H,0} exp\left(\frac{\Delta U_{L/U/H}}{RT}\right)$
		$w = \left(exp\left(\frac{\left(log\left(y_{\mathrm{CO_2}} p\right)-log\left(p_{step}\right)\right)}{\sigma}\right) / \left(1+exp\left(\frac{\left(log\left(y_{\mathrm{CO_2}} p\right)-log\left(p_{step}\right)\right)}{\sigma}\right)\right)\right)^{\gamma}$
		$p_{step} = p_{step,0} exp\left(\frac{-\Delta H_{step}}{R}\left(\frac{1}{T0}-\frac{1}{T}\right)\right)$
		$\sigma = Xi_1 * exp\left(Xi_2\left(\frac{1}{T_0}-\frac{1}{T}\right)\right)$, $q_U = \frac{q_{U,0} * b_U\left(y_{\mathrm{CO_2}} p\right)}{1+b_U\left(y_{\mathrm{CO_2}} p\right)} + b_H\left(y_{\mathrm{CO_2}} p\right)$

The NIST/ARPA-E database, the database of novel and emerging adsorbents by the National Institute of Standards and Technology (NIST) includes data from published studies, both real and hypothetical. Since the database includes thousands of isotherm data files, not only for CO_2 adsorption, but also many other gases, it is important to first filter the materials. We use a script written in MATLAB to run through the materials filter the suitable adsorbents. The approach is graphically shown in Figure S9. During the filtering process, we are also excluding all data sets for which only one temperature is available. This is necessary for the following step, where the remaining isotherms are fitted by temperature dependent isotherm models. The fitting process itself includes several steps, which are shown in the flowchart in Figure S10. For the objective function during the fitting, the normalized standard deviation was applied, which is commonly used to fit isotherm models to experimental data [4–7]. It includes the adsorbed amount determined experimentally q_{exp}, the amount adsorbed as predicted by the model q_{fit} and the total number of experimental points N, and is calculated in the form of

$$err = \sqrt{\frac{\sum\left[\frac{(q_{\text{exp}}-q_{\text{fit}})}{q_{\text{exp}}}\right]^2}{N-1}} \cdot 100 \tag{S37}$$

which is minimized using the inbuilt Matlab optimization routine *fmincon* together with the *sequential quadratic programming (SQP)* algorithm.

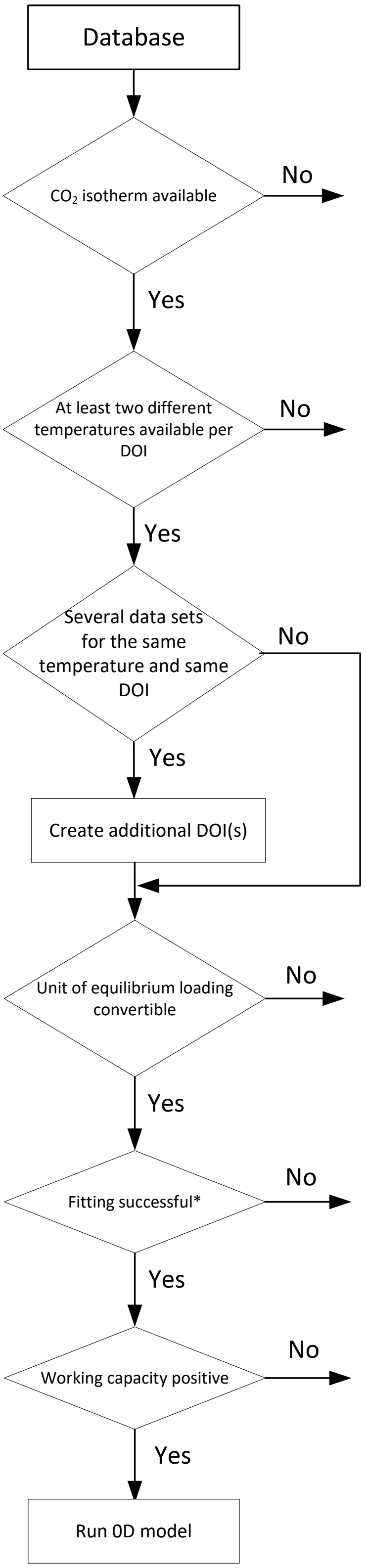

Figure S9: Flowchart describing the filtering process of the adsorbents from the NIST/ARPA-E database.

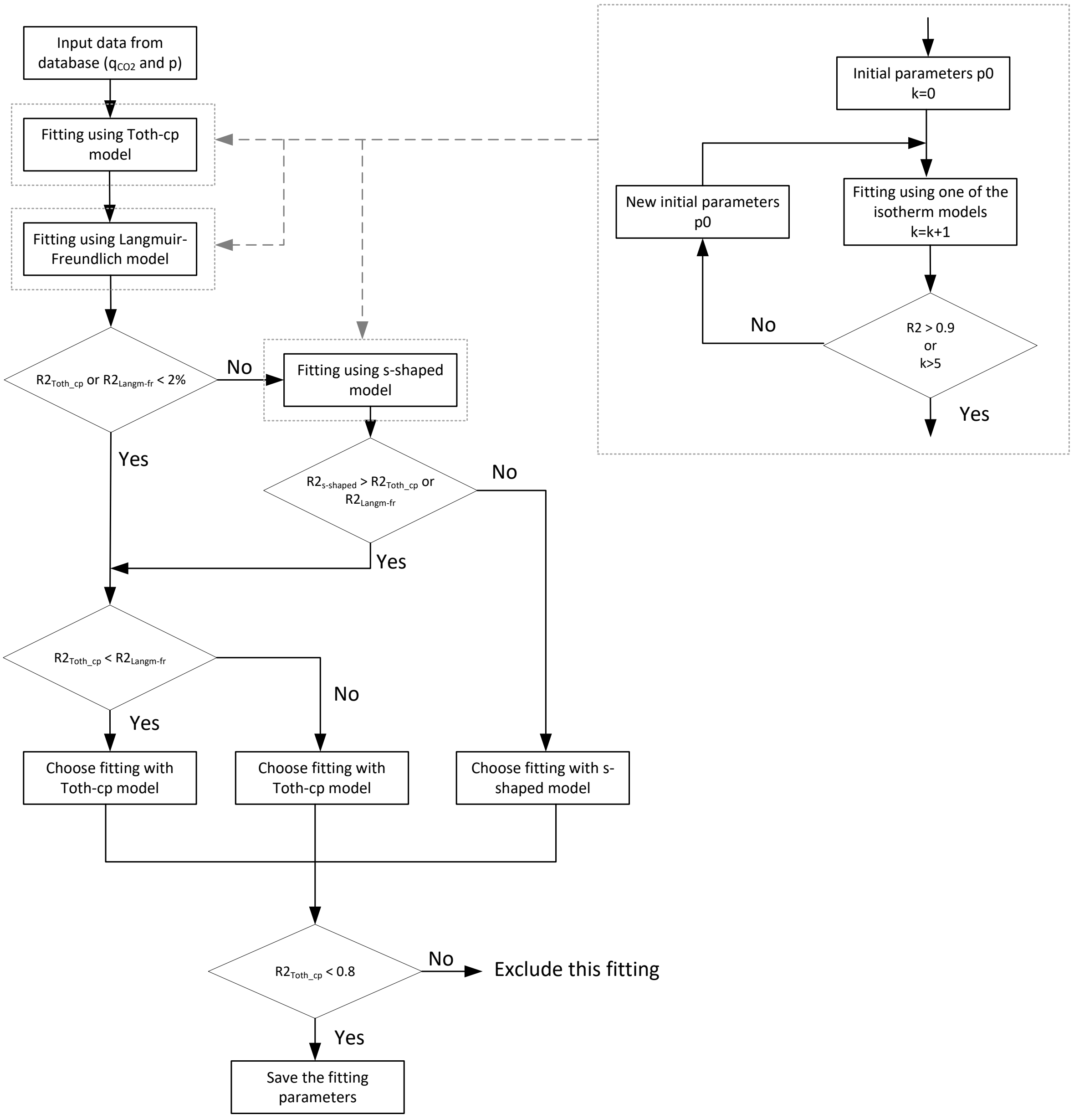

Figure S10: Flowchart fitting.

The error is expressed by

$$R2 = \frac{\sum \left(q_{\text{exp}} - q_{\text{fit}}\right)^2}{\sum \left(q_{\text{exp}} - \overline{q_{\text{exp}}}\right)^2} \tag{S38}$$

with $\overline{q_{\text{exp}}}$ being the mean of the experimental data.

Table S13: Resulting ranking showing the 10 best performing materials for $y_{CO_2} = 0.4\%$. The values of the maximum productivity and the corresponding thermal energy consumption are given for the best performing adsorbent. For the remaining materials, the deviation to the best performing one is given in percentage.

	Adsorbent	Pr	Q_{th}
1	Cr-MIL(101)	$20.6\,\mathrm{kg/m^3/h}$	$14.4\,\mathrm{MJ/kg}$
2	MIL-101	-5%	-4%
3	CuBTC	-24%	+9%
4	MIL-53(Al)	-10%	+42%
5	Zn-DABCO	-24%	+49%
6	MIL-101(Cr)-PEI-800	-66%	-11%
7	Lewatit	-74%	+9%
8	Exemplary	-77%	+18%
9	Zeolite Na-LSX	-21%	+61%
10	Ca-X	-27%	+52%

Table S14: Resulting ranking showing the 10 best performing materials for $y_{CO_2} = 0.1\%$. The values of the maximum productivity and the corresponding thermal energy consumption are given for the best performing adsorbent. For the remaining materials, the deviation to the best performing one is given in percentage.

	Adsorbent	Pr	Q_{th}
1	PCN-11	$43.8\,\mathrm{kg/m^3/h}$	$14.9\,\mathrm{MJ/kg}$
2	MIL-101	-8%	-2%
3	Cr-MIL(101)	0%	+33%
4	Ca-X	-25%	+12%
5	Zeolite Na-LSX	-32%	+32%
6	Cu-BTC powder	-31%	+52%
7	CuBTC	-29%	+54%
8	MIL-53(Al)	-15%	+96%
9	Zn-DABCO	-26%	+53%
10	MIL-101(Cr)-PEI-800	-71%	-30%

Table S15: Resulting ranking showing the 10 best performing materials for $y_{CO_2} = 1.0\%$. The values of the maximum productivity and the corresponding thermal energy consumption are given for the best performing adsorbent. For the remaining materials, the deviation to the best performing one is given in percentage.

	Adsorbent	Pr	Q_{th}
1	Mg-MOF-74	$207.7\,\mathrm{kg/m^3/h}$	$4.72\,\mathrm{MJ/kg}$
2	Ca-X	-32%	+68%
3	MIL-101(Cr)-250nm-PEI-300	-36%	+88%
4	Zeolite Na-LSX	-47%	+118%
5	Zeolite 13X	-49%	+106%
6	PCN-11	-41%	+205%
7	MIL-101	-52%	+206%
8	Carbon	-54%	+139%
9	Cr-MIL(101)	-43%	+318%
10	Mg-X	-60%	+164%

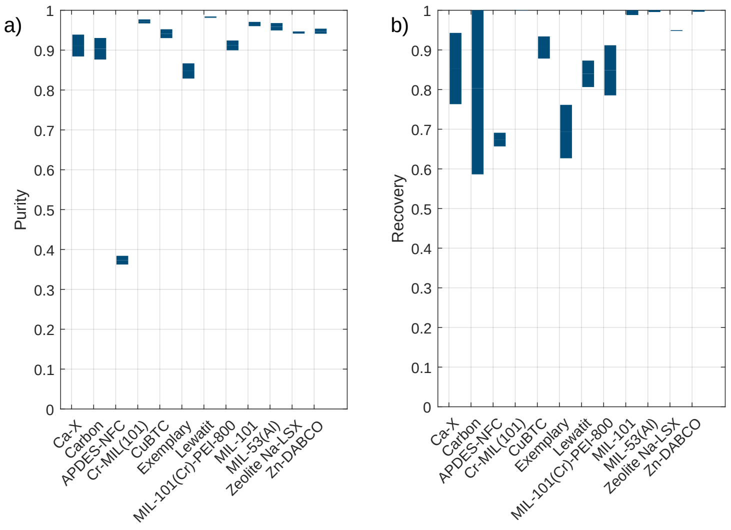

Figure S11: a) Purity and b) recovery for resulting materials from screening with 0.04% CO_2 in the feed.

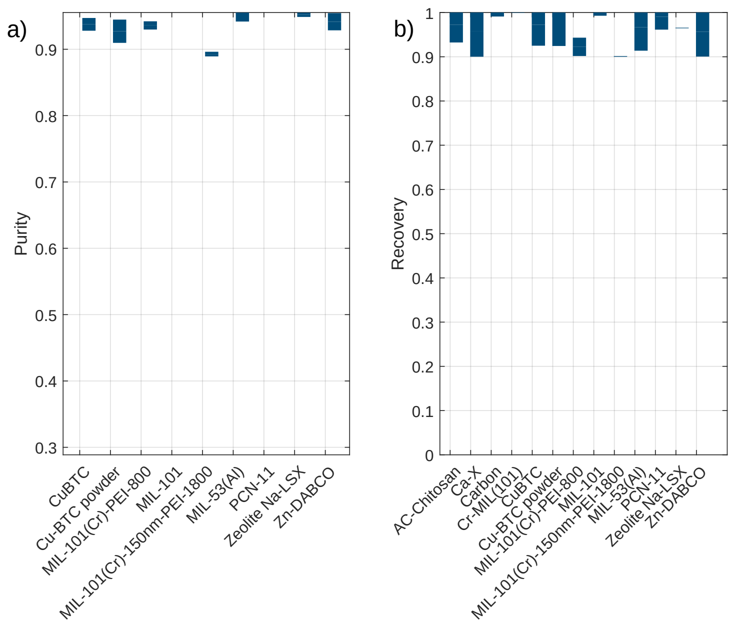

Figure S12: a) Purity and b) recovery for resulting materials from screening with 0.1% CO_2 in the feed.

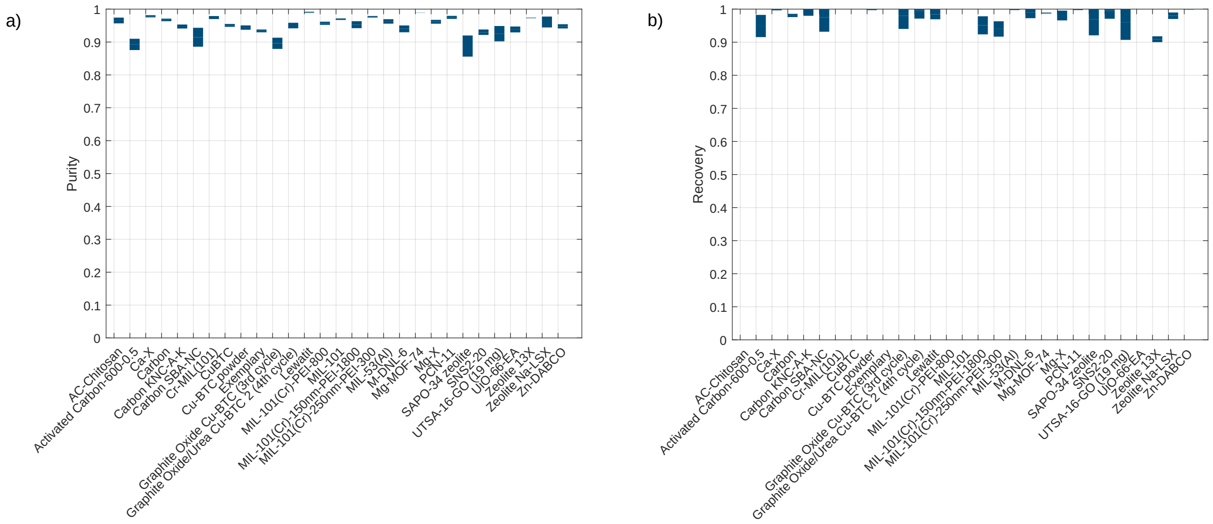

Figure S13: a) Purity and b) recovery for resulting materials from screening with 1.0% CO_2 in the feed.

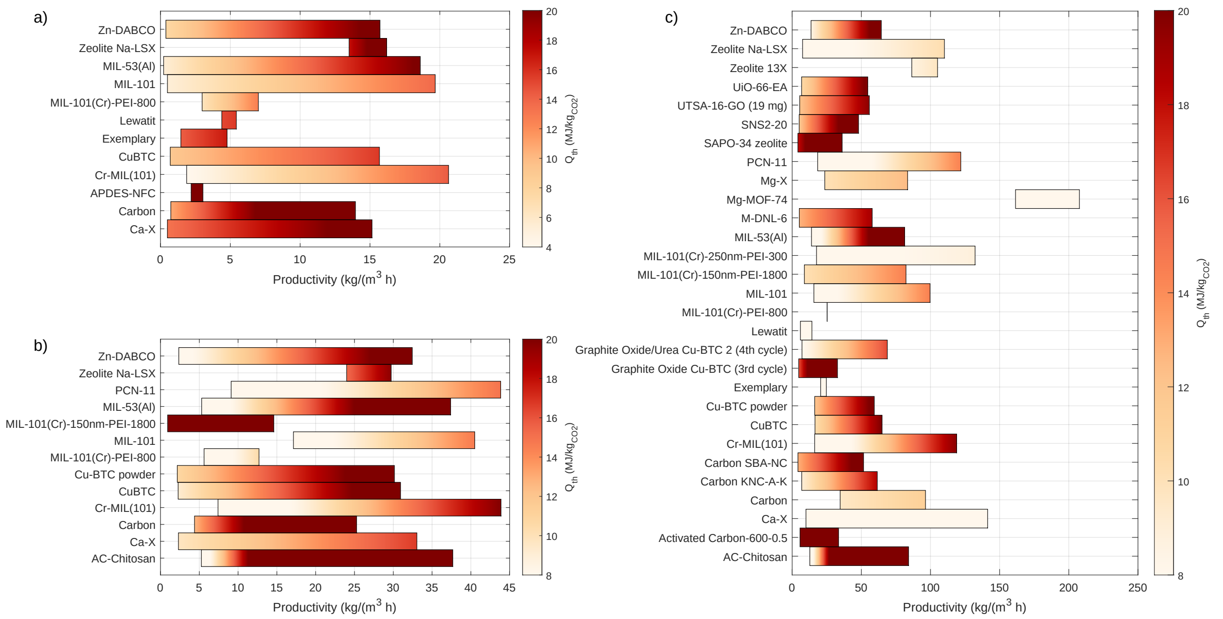

Figure S14: Resulting objectives humid using Lewatit H_2O isotherm for a) 0.04%, b) 0.1% and c) 1.0% CO_2 in the feed.

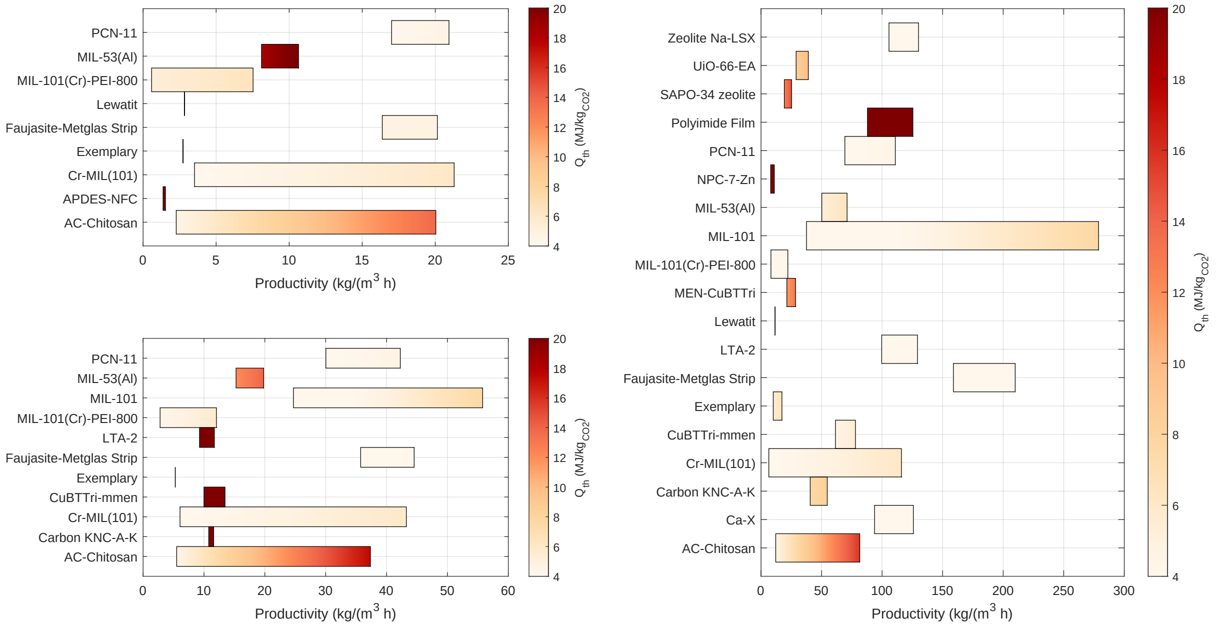

Figure S15: Resulting objectives with dry feed for a) 0.04%, b) 0.1% and c) 1.0% CO_2 in the feed.

The following three Tables S16, S17, and S18 list the resulting materials from the screening with different CO_2 concentrations for the case with the APDES-NFC isotherm for water.

Table S16: Resulting materials with their DOI from screening with $y_{CO_2} = 40Pa$ and the APDES-NFC isotherm for the water.

Material	DOI
Ca-X	10.1039/c2ee23337a
Carbon	10.1021/la4004998
APDES-NFC	10.1021/es404430g
Cr-MIL(101)	10.1039/C2ra20641b
CuBTC	10.1016/j.cej.2015.07.020
Exemplary	10.1016/j.joule.2021.05.023
Lewatit	10.1016/j.ijggc.2015.07.014
MIL-101(Cr)-PEI-800	10.1021/acssuschemeng.6b01692
MIL-101	10.1021/Jp811418r
MIL-53(Al)	10.1021/Ie5006146
Zeolite Na-LSX	10.1016/S0167-2991(02)80202-6
Zn-DABCO	10.1016/j.seppur.2011.09.041

Table S17: Resulting materials with their DOI from screening with $y_{CO_2} = 100Pa$ and the APDES-NFC isotherm for the water.

**Material**	**DOI**
AC-Chitosan	10.1016/j.physe.2013.10.024
Ca-X	10.1039/c2ee23337a
Carbon	10.1021/la4004998
Cr-MIL(101)	10.1039/C2ra20641b
CuBTC	10.1016/j.cej.2015.07.020
Exemplary	10.1016/j.joule.2021.05.023
Lewatit	10.1016/j.ijggc.2015.07.014
MIL-101(Cr)-PEI-800	10.1021/acssuschemeng.6b01692
MIL-101	10.1021/Jp811418r
MIL-101(Cr)-150nm-PEI-1800	10.1039/C4ta01174k
MIL-53(Al)	10.1021/Ie5006146
PCN-11	10.1039/C0ee00700e
Zeolite Na-LSX	10.1016/S0167-2991(02)80202-6
Zn-DABCO	10.1016/j.seppur.2011.09.041

Table S18: Resulting materials with their DOI from screening with $y_{CO_2} = 1000Pa$ and the APDES-NFC isotherm for the water.

**Material**	**DOI**
AC-Chitosan	10.1016/j.physe.2013.10.024
Activated Carbon-600-0.5	10.1039/C2cp44436d
Ca-X	10.1039/c2ee23337a
Carbon	10.1021/la4004998
Carbon KNC-A-K	10.1021/Cm303072n
Carbon SBA-NC	10.1021/Cm303072n
Cr-MIL(101)	10.1039/C2ra20641b
CuBTC	10.1016/j.cej.2015.07.020
Exemplary	10.1016/j.joule.2021.05.023
Graphite Oxide Cu-BTC (3rd cycle)	10.1021/am404952z
Graphite Oxide/Urea Cu-BTC 2 (4th cycle)	10.1021/am404952z
Lewatit	10.1016/j.ijggc.2015.07.014
MIL-101(Cr)-PEI-800	10.1021/acssuschemeng.6b01692
MIL-101	10.1021/Jp811418r
MIL-101(Cr)-150nm-PEI-1800	10.1039/C4ta01174k
MIL-101(Cr)-250nm-PEI-300	10.1039/C4ta01174k
MIL-53(Al)	10.1021/Ie5006146
M-DNL-6	10.1002/cssc.201200907
Mg-MOF-74	10.1016/j.ijggc.2013.01.009
Mg-X	10.1039/c2ee23337a
PCN-11	10.1039/C0ee00700e
SAPO-34 zeolite	10.1080/01496395.2013.812118
SNS2-20	10.1039/c5ta01776a
UTSA-16-GO (19 mg)	10.1039/c4ta04770b
UiO-66-EA	10.1039/c5ta05997f
Zeolite 13X	10.1021/Je800900a
Zeolite Na-LSX	10.1016/S0167-2991(02)80202-6
Zn-DABCO	10.1016/j.seppur.2011.09.041

Nomenclature

ρ density, $\mathrm{kg/m^3}$

α saturation level

$\Delta \mathrm{H_{ads}}$ isosteric heat of adsorption, $\mathrm{J/mol}$

$\Delta \mathrm{H_{ads}}$ isosteric heat of adsorption, $\mathrm{kJ/mol}$

$\dot{\mathrm{Q}}$ specific heat, $\mathrm{kJ/kg}$

$\dot{\mathrm{V}}_{\mathrm{feed}}$ feed volume stream, $\mathrm{m^3/s}$

ϵ void fraction

μ dynamic viscosity, Pas

Φ purity

$\mathrm{c_p}$ specific heat capacity at constant pressure, $\mathrm{kJ/kg/K}$

$\mathrm{C_{ads}}$ heat capacity of the adsorbed phase, $\mathrm{J/(Kkg)}$

$\mathrm{C_g}$ heat capacity of the gas, $\mathrm{J/(Km^3)}$

$\mathrm{C_s}$ heat capacity of the solid, $\mathrm{J/(Kkg)}$

$\mathrm{d_p}$ particle diameter, m

$\mathrm{e^{th}}$ Specific thermal energy, $\mathrm{kJ/kg_{CO_2}}$

h heat transfer coefficient, $\mathrm{J/(m^2sK)}$

k overall mass transfer coefficient, $\mathrm{1/s}$

$\mathrm{N_{grid}}$ number of grid points

r capture rate

$\mathrm{u_{air}}$ air velocity, $\mathrm{m/s}$

N amount of moles, mol

$R2$ error

a,b,c,d,e,f,g,h fitting parameters

ads adsorption

amb ambient

BD blow-down

des desorption

err error

exp experimental

f fluid,final

fit fitted

i component

m mass, kg

MM Molar mass, $\mathrm{g/mol}$

p pressure, bar

Pr Productivity, $\mathrm{kg/m^3/h}$

q solid phase concentration, $\mathrm{mol/kg}$

q^* equilibrium adsorbed amount, $\mathrm{mol/kg}$

R universal gas constant, $\mathrm{J/mol/K}$

s solid,sorbent

T temperature, K

t time, s

V_c column volume, $\mathrm{m^3}$

vac vacuum

y mole fraction

References

[1] R. Douglas M., *Principles of Adsorption and Adsorption Processes*. New York: John Wiley & Sons, 1984.

[2] L. Joss, M. Gazzani, and M. Mazzotti, "Rational design of temperature swing adsorption cycles for post-combustion CO2 capture," *Chemical Engineering Science*, vol. 158, pp. 381–394, feb 2017.

[3] F. Sabatino, A. Grimm, F. Gallucci, M. van Sint Annaland, G. J. Kramer, and M. Gazzani, "A comparative energy and costs assessment and optimization for direct air capture technologies," *Joule*, vol. 5, pp. 2047–2076, aug 2021.

[4] C. Gebald, N. Platkowskl, T. Rüesch, and J. A. Wurzbacher, "Low-pressure drop structure of particle adsorbent bed for gas adsorption separation process," apr 2014. Accessed: 05.07.2022.

[5] J. Elfving, C. Bajamundi, J. Kauppinen, and T. Sainio, "Modelling of equilibrium working capacity of PSA, TSA and TVSA processes for CO2 adsorption under direct air capture conditions," *Journal of CO2 Utilization*, vol. 22, pp. 270–277, dec 2017.

[6] Y. Lin, Q. Yan, C. Kong, and L. Chen, "Polyethyleneimine incorporated metal-organic frameworks adsorbent for highly selective CO2 capture," *Scientific Reports*, vol. 3, 2013.

[7] R. Serna-Guerrero, Y. Belmabkhout, and A. Sayari, "Modeling CO2 adsorption on amine-functionalized mesoporous silica: 1. A semi-empirical equilibrium model," *Chemical Engineering Journal*, vol. 161, no. 1-2, pp. 173–181, 2010.
